# Heterosis and Hybrid Crop Breeding: A Multidisciplinary Review

**DOI:** 10.3389/fgene.2021.643761

**Published:** 2021-02-24

**Authors:** Marlee R. Labroo, Anthony J. Studer, Jessica E. Rutkoski

**Affiliations:** Department of Crop Sciences, University of Illinois at Urbana–Champaign, Urbana, IL, United States

**Keywords:** heterosis, inbreeding depression, genomic selection, reciprocal recurrent genomic selection, dominance, autogamous

## Abstract

Although hybrid crop varieties are among the most popular agricultural innovations, the rationale for hybrid crop breeding is sometimes misunderstood. Hybrid breeding is slower and more resource-intensive than inbred breeding, but it allows systematic improvement of a population by recurrent selection and exploitation of heterosis simultaneously. Inbred parental lines can identically reproduce both themselves and their F_1_ progeny indefinitely, whereas outbred lines cannot, so uniform outbred lines must be bred indirectly through their inbred parents to harness heterosis. Heterosis is an expected consequence of whole-genome non-additive effects at the population level over evolutionary time. Understanding heterosis from the perspective of molecular genetic mechanisms alone may be elusive, because heterosis is likely an emergent property of populations. Hybrid breeding is a process of recurrent population improvement to maximize hybrid performance. Hybrid breeding is not maximization of heterosis *per se*, nor testing random combinations of individuals to find an exceptional hybrid, nor using heterosis in place of population improvement. Though there are methods to harness heterosis other than hybrid breeding, such as use of open-pollinated varieties or clonal propagation, they are not currently suitable for all crops or production environments. The use of genomic selection can decrease cycle time and costs in hybrid breeding, particularly by rapidly establishing heterotic pools, reducing testcrossing, and limiting the loss of genetic variance. Open questions in optimal use of genomic selection in hybrid crop breeding programs remain, such as how to choose founders of heterotic pools, the importance of dominance effects in genomic prediction, the necessary frequency of updating the training set with phenotypic information, and how to maintain genetic variance and prevent fixation of deleterious alleles.

## Introduction

Hybrid crop varieties vastly outperform their inbred progenitors in economically important species ranging from maize (*Zea mays*) to oil palm (*Elaeis guineensis*; Duvick, 2005; Fu et al., 2014; Cros et al., 2015). However, hybrid breeding requires more time and resources than inbred breeding (Troyer and Wellin, 2009; Longin et al., 2014; Cros et al., 2018). The effectiveness of hybrid breeding can be improved by genomic selection, in which marker information partially replaces phenotypes in estimation of breeding values (Heffner et al., 2009). Genomic selection can shorten the breeding cycle, reduce the costs of phenotyping, and improve selection accuracies (Lorenz et al., 2011; Heslot et al., 2015; Zhao et al., 2015b; Schulthess et al., 2017; Kadam and Lorenz, 2018). Genomic selection also opens new opportunities to establish hybrid breeding programs in crops which are widely cultivated as inbreds, such as wheat (*Triticum aestivum*; Zhao et al., 2015b). Here, we compare and contrast genomic selection with conventional selection in hybrid crop breeding. We summarize the quantitative genetic model of phenotype, and we synthesize quantitative, evolutionary, phenotypic, and molecular genetic perspectives to explain the bases of heterosis and its role in breeding hybrids. Then, we cover the fundamentals of genomic prediction and its uses in genomic selection at all stages of the hybrid breeding cycle, including selection strategies for long-term gain. In closing, we outline factors which influence the success of hybrid breeding programs relative to inbred breeding programs.

## Quantitative Genetic Model of Phenotype

To consider genomic selection for hybrid performance and heterosis, it is necessary to understand the statistical model of phenotype used in quantitative genetics. The observed performances of individuals in a population are their phenotypic values (Falconer and Mackay, 1996). The variance of individuals’ phenotypic values is due to genetic and non-genetic variance components and their interactions (Supplementary Table 1; Eq. 1; Falconer and Mackay, 1996). If non-genetic variance were absent, then phenotypic variance would be equal to genetic variance. Detecting genetic variance does not require demonstrating molecular modes of gene action, and genetic effects are indirectly observed as differences in phenotypes (Falconer and Mackay, 1996). For example, if there are no differences in individuals’ phenotypes and thus no phenotypic variance, then genetic effects and genetic variance are zero. Even though at the molecular genetic level cellular machinery dynamically generates and maintains identical phenotypes, these are not genetic effects or genetic variance in the quantitative genetic sense. Similarly, the amount of genetic variation, genetic diversity, or nucleotide diversity cannot be inferred from the magnitude of genetic variance even though genetic variation underlies genetic variance. If the most genetically diverse lines of a population are sampled and their phenotypes are identical, then genetic variance is nonetheless zero, assuming no non-genetic variance. If the phenotype is also measured in closely related lines but varies greatly, then genetic variance is large, even if the lines have nucleotide polymorphisms in just one gene.

Total genetic variance can be further partitioned into additive, dominance, and epistatic variance (Supplementary Table 1; Eq. 2; Falconer and Mackay, 1996). Intuitively, individuals share alleles to the degree that they are related (Falconer and Mackay, 1996; Fisher, 1918). Under the infinitesimal model, an impossibly large number of alleles additively affect quantitative trait phenotypes, so the proportion of shared alleles among relatives is expected to produce concomitant phenotypic resemblance (Fisher, 1918). The more that relatives phenotypically resemble each other in proportion to their degree of relatedness, the greater the proportion of phenotypic variance that can be explained by additive genetic variance, assuming zero non-genetic variance (Fisher, 1918). If dominance and epistatic variance is present, relatives may resemble each other more than expected by a strictly additive model (Lynch and Walsh, 1998).

Genetic variance is also viewed as statistical effects of alleles at individual loci in a population (Falconer and Mackay, 1996). Alleles can have additive and dominance effects on genetic value. At a given locus, the additive effect of an allele, *a*, is the average genetic value of all individuals which are homozygous for the allele (Falconer and Mackay, 1996). The dominance effect of the allele, *d*, is the average genetic value of all individuals which are heterozygous for the allele (Falconer and Mackay, 1996). Since epistasis requires multiple alleles, single alleles do not have epistatic effects.

At the population level, the average effect of substituting one allele for another at a given locus on the genetic mean of the population depends not only on the additive and dominance effects of the allele, but also its frequency (Supplementary Table 1, Eq. 3—5; Falconer and Mackay, 1996). The average effect of an allele, α, is its coefficient in regression of genetic value on the number of copies of the allele in each genotype at the locus (Supplementary Table 1; Eq. 6; Falconer and Mackay, 1996). If dominance occurs, then observed genetic values do not fall exactly on the regression line of genetic value on allele copy number per genotype (Falconer and Mackay, 1996). The deviation of the heterozygote genetic value from the regression line is the dominance deviation of the allele, δ (Supplementary Table 1; Eq. 6; Falconer and Mackay, 1996). If more than one locus affects phenotype, then epistatic interactions between and/or among allelic effects across loci can also contribute to genetic value (Supplementary Table 1; Eq. 7; Falconer and Mackay, 1996). The statistical effects of alleles can be used directly to calculate respective genetic variances, but realistically it is almost always unknown which alleles affect phenotype or which individuals carry which alleles (Falconer and Mackay, 1996). Therefore, in practice, genetic variances are estimated from resemblance among relatives, not *a priori* from allelic effects (Falconer and Mackay, 1996).

Statistical genetic average, dominance, and epistatic effects do not represent underlying biological modes of gene action in most experimental and breeding settings, and modes of gene action cannot be inferred from the relative contribution of each source of statistical genetic effects to the genetic value (Cordell, 2002; Crow, 2010; de los Campos et al., 2015; Huang and Mackay, 2016; Manfredi et al., 2017). By definition, biologically dominant or epistatic gene action is largely captured by statistical average effects, because statistical dominance and epistasis are residual deviations from average effects (Cheverud and Routman, 1995). Average, dominance, and epistatic effects refer to their statistical formulations throughout this review unless specified as biological.

## Quantitative Genetic Model of Heterosis

A rationale for hybrid breeding is the systematic exploitation of heterosis (Schulthess et al., 2017). Mid-parent heterosis is the difference between a progeny genetic value and its mid-parent value, or the average of its parents’ genetic values (Supplementary Table 1.; Eq. 8; Lynch and Walsh, 1998). Under the additive model, the genetic value of a progeny is expected to be equal to the average genetic value of its parents. Thus, mid-parent heterosis results from dominance and epistatic deviation. However, mid-parent heterosis of a single cross is neither a measure of dominance or epistatic effects nor a measure of heterosis in a population.

**FIGURE 1:**
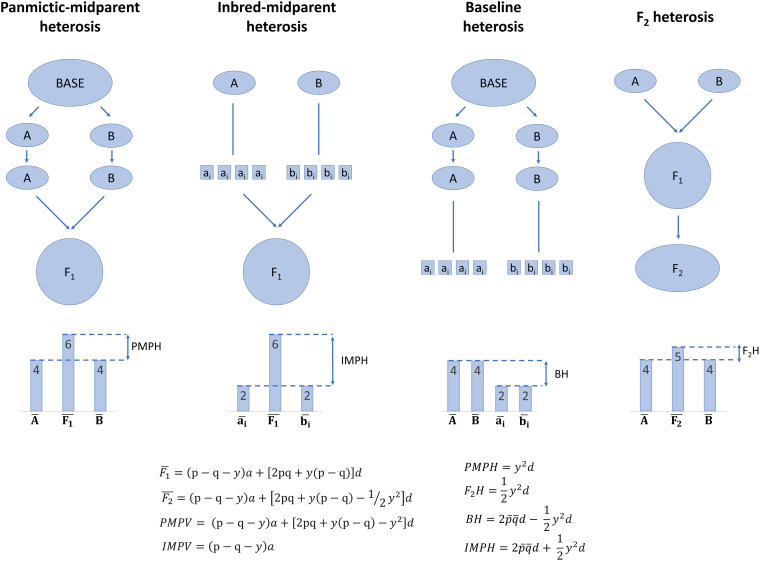
Illustration of the partitions of population-level heterosis by Lamkey and Edwards (1999). Arrows indicate random mating or random crossing, and lines indicate selfing to homozygosity. Note that only dominance, additive × dominance, and dominance × dominance effects can contribute to baseline heterosis, but dominance, additive × dominance, dominance × dominance, and additive × additive effects can contribute to panmictic-midparent heterosis, inbred-midparent heterosis, and F_2_ heterosis. Equations are further described in Supplementary Table 1.

It is important to define heterosis further at the population level, because (a) heterosis emerges at the population level, even if it partially can be observed in single crosses, and (b) breeding involves populations rather than individuals alone (Figure 1; Lamkey and Edwards, 1999). In a group of individuals which can potentially intermate, such as a species, random mating may not occur among all individuals. Non-random mating of individuals—or any factor which leads to Hardy–Weinberg disequilibrium, such as migration—can cause distinct subpopulations form within the overall population, termed population structure. Within subpopulations, mating is random and Hardy–Weinberg equilibrium is reached, but among subpopulations mating is non-random. The subpopulations are inbred relative to the population that would result if random mating had occurred among all individuals in the overall population, and allele frequencies may come to differ among subpopulations.

What results if two of these subpopulations are randomly mated to each other? The mean genetic value of their F_1_ may differ from the mean of the average genetic values within each subpopulation, and this difference is termed panmictic-midparent heterosis (Supplementary Table 1; Eq. 9—11; Lamkey and Edwards, 1999). Panmictic-midparent heterosis is thought to result from (a) dominance, as allele frequencies differ between the parent populations, and dominant genotypes that do not occur in the parents are observed in their F_1_, and/or (b) additive × additive epistasis, as new interactions among alleles are possible in the F_1_ compared to the parents. The portion of panmictic-midparent heterosis due to dominance, if present, can be thought of as recovery from inbreeding due to population structure, because subpopulations are by definition inbred relative to a population in which structure had never occurred. Although this base population in which structure never occurred is hypothetical and cannot be observed, it is possible to form an analogous population in Hardy–Weinberg equilibrium by randomly intermating the two subpopulations to form an F_1_, then randomly mating the F_1_ to form an F_2_. Heterosis in the F_2_, or F_2_ heterosis, is reduced by half compared to the panmictic-midparent heterosis in the F_1_ (Supplementary Table 1; Eq. 12—13; Lamkey and Edwards, 1999).

Panmictic-midparent heterosis can be positive or negative. If panmictic-midparent heterosis is negative, it is sometimes referred to as outbreeding depression (Waser and Price, 1994; Lynch and Walsh, 1998; Grindeland, 2008; Oakley et al., 2015). Outbreeding depression is thought to primarily result not from dominance, but rather from loss of favorable additive × additive epistases as co-selected, genomically compatible combinations of alleles are separated in the F_1_ of two random-mating populations (Dobzhansky, 1941; Welch, 2004). These losses of favorable biological epistases are termed Dobzhansky–Muller incompatibilities (Dobzhansky, 1941).

What results if, within a subpopulation, inbreeding occurs rather than random mating? Inbred lines are often observed to have a lower mean genetic value than the mean of the less inbred subpopulation, a phenomenon referred to as inbreeding depression (Charlesworth and Charlesworth, 1987; Falconer and Mackay, 1996; Charlesworth and Willis, 2009). Inbreeding depression is thought to result biologically from (a) deleterious recessive alleles driven to homozygosity, (b) homozygosity at overdominant loci at which the heterozygous state outperforms either homozygote, and/or (c) a loss of favorable epistatic interactions between heterozygous genotypes (Davenport, 1908; East, 1908; Shull, 1908; Falconer and Mackay, 1996). If it were possible to randomly mate the inbred lines without selection to form an F_1_, the original subpopulation would be reconstituted and its mean restored to its original state if inbreeding depression were due to dominance and/or epistasis (Falconer and Mackay, 1996). Interestingly, there is some evidence of inbreeding depression due to epigenetic changes which may not be reversible by random mating, termed hybrid decay or hybrid dysgenesis (de la Luz Gutiérrez-Nava et al., 1998; Xue et al., 2019). Though hybrid decay is not thought to be a universal cause of inbreeding depression and has not prevented production of hybrids from inbreds in commercial programs, it is unknown how widespread this occurrence is.

If two subpopulations are again considered, an F_1_ of the subpopulations can be produced in two ways: (a) the randomly mating subpopulations can be randomly intermated, or (b) each subpopulation can first be selfed to produce fully inbred lines, and the fully inbred lines can be randomly intermated (Lamkey and Edwards, 1999). The average genetic value of the F_1_ resulting from either of these processes is equal (Supplementary Table 1; Eq. 9; Lamkey and Edwards, 1999). Some of the mean genetic value of the F_1_ is due to baseline heterosis, or the restoration of what was lost due to inbreeding depression during selfing of both the parent subpopulations (Supplementary Table 1; Eq. 14; Lamkey and Edwards, 1999). However, the panmictic-midparent heterosis that results from crossing two randomly mating subpopulations also contributes to the genetic value of these F_1_. Therefore, inbred-midparent heterosis is defined as the sum of baseline heterosis and panmictic-midparent heterosis, which is equivalent to the difference of the mean genetic value of the F_1_ and the mean genetic value of all the inbred parents derived from both populations (Supplementary Table 1; Eq. 9, Eq. 15—16; Lamkey and Edwards, 1999). The key reason to partition heterosis into panmictic-midparent, F_2_, baseline, and inbred-midparent heterosis is that it allows definition of average heterosis and inbreeding depression at the population level. Furthermore, it provides a framework to contrast heterosis that results from crossing two random-mating subpopulations and heterosis that results from crossing inbred lines that result from the random-mating subpopulations.

Heterosis is often described as the “opposite” or converse of inbreeding depression. However, of the partitions of heterosis described here, only baseline heterosis is strictly the opposite of inbreeding depression. Panmictic-midparent heterosis (and therefore inbred-midparent heterosis) can arise totally from epistatic effects without dominance, whereas inbreeding depression cannot (Falconer and Mackay, 1996; Lamkey and Edwards, 1999; Chen, 2013). Heterosis due to epistasis can only result from additive × additive epistasis, whereas inbreeding depression can result from both additive × dominance and dominance × dominance epistasis (Lynch, 1991; Lynch and Walsh, 1998).

## Evolutionary and Molecular Genetic Bases of Heterosis

From the evolutionary perspective, heterosis in quantitative genetics ultimately rests on assumptions of biological dominance and biological epistasis, even though the additive model captures most of the effects of biological dominance and epistasis (Huang and Mackay, 2016). For biological dominance to affect heterosis, dominant alleles should have directional effects on fitness (Lynch and Walsh, 1998). As biologically dominant mutations arise in a population, they affect phenotype regardless of zygosity and are exposed to selection (Falconer and Mackay, 1996). Recessive mutations are only exposed to selection on phenotype in their homozygous state and can propagate in populations as they are not selected as heterozygotes (Falconer and Mackay, 1996). Therefore, deleterious dominant alleles are more likely to be eliminated from populations by selection than deleterious recessives (Falconer and Mackay, 1996). Over evolutionary time, it is expected that biologically dominant alleles tend to be favorable, and deleterious alleles tend to be recessive (Falconer and Mackay, 1996). If dominant alleles have directional effects on fitness, the effect is then often positive (i.e., the sign of dominance occurs in the same direction as the measure of fitness). In maize, alleles identified as likely deleterious via genomic evolutionary rate profiling were found more likely to be recessive (Yang et al., 2017). The likelihood of purging recessive deleterious alleles is reduced as effective population size increases, and deleterious alleles may be shielded from selection in genomic regions with low recombination rates, such as the centromere, regardless of dominance (Barrett and Charlesworth, 1991; Rodgers-Melnick et al., 2015; Yang et al., 2017). Evolutionary mechanisms besides directional selection have also been proposed to explain the emergence of dominance, such as stabilizing selection (Manna et al., 2011).

Heterosis can also result from overdominance, a type of biological dominance in which heterozygotes have more extreme phenotypes than both homozygotes, and alleles persist in populations at intermediate frequencies (Crow, 1999). One overdominant locus alone is sufficient to cause heterosis (Falconer and Mackay, 1996; Krieger et al., 2010). However, detection of overdominance is complicated because it requires inbred parents to be identical at all loci except the locus of interest. If they are not, then parents can carry biologically dominant alleles of opposite effects on fitness linked in repulsion, and pseudooverdominance results: the loci are never observed in their uncoupled state, and they appear as one overdominant locus. In absence of linkage, pseudooverdominance would not exist.

Finally, biological epistasis may contribute to heterosis as interactions of multiple loci contribute to fitness. Ample evidence of biological epistasis is available; for example, genes encoding transcription factor proteins may physically bind to DNA sequence motifs to activate or repress other genes which affect phenotype, among other mechanisms (Phillips, 2008; Lehner, 2011; Burdo et al., 2014). However, detecting all types of both statistical and biological epistasis in regular experimental samples is often not feasible because the number of combinations of alleles is much larger than the number of individual genotypes in a population (Wei et al., 2014). Epistasis also cannot be detected in a population if the experimental sample is not segregating for both interacting genes (Stitzer and Ross-Ibarra, 2018).

It is possible that heterosis can be explained fully by biologically additive, dominant, and epistatic gene action and that no single gene, class of genes, or physiological phenomenon causes heterosis (Birchler et al., 2010; Fiévet et al., 2018). If so, searching for the genetic basis of heterosis would lead to the genetic basis of the specific trait in question in a particular experimental sample, and heterosis would be conferred by biological dominance, overdominance, or epistasis of those genes which controlled the trait (Fiévet et al., 2018). For example, consider inquiry into the genetic basis of heterosis for grain yield in maize and biomass yield in sorghum (*Sorghum bicolor*). By the explanation of heterosis above, maize grain yield and sorghum biomass yield could be controlled by completely different genes and classes of genes, and dominant, overdominant, or epistatic action of the genes involved would lead to heterosis. If more individuals were sampled, which presented more combinations of genes and/or more genetic variants, then the genetic basis of observed heterosis could change.

It has been further hypothesized that actions of particular classes of genes or physiological effects of genes cause heterosis universally across traits and species (Birchler et al., 2010; Fiévet et al., 2018). These proposed unifying mechanisms include organellar complementation, circadian rhythm changes, changes in hormone expression, genome-wide changes in chromatin state and/or changes in small RNA expression, dosage effects, regulatory incompatibility, parent-specific gene expression, and changes in signaling in response to heterozygosity (Auger et al., 2005; Reif et al., 2005; Lippman and Zamir, 2007; Kaeppler, 2012; Chen and Birchler, 2013; Bar-Zvi et al., 2017; Herbst et al., 2017; Li et al., 2020). It is challenging to detangle whether each of these actions of gene classes and physiological effects are themselves causes of heterosis, or instead the effect of a true unobserved cause of heterosis. None has been demonstrated to universally explain heterosis, but some have been demonstrated to be associated with heterosis in some cases and have been incorporated into predictive models with varying effects on prediction accuracy (Kaeppler, 2012; Westhues et al., 2017; Schrag et al., 2018; Seifert et al., 2018). At the transcriptome level, hybrids generally display transcript levels near their mid-parent value, with some exceptions (Swanson-Wagner et al., 2006; Hochholdinger and Hoecker, 2007; Springer and Stupar, 2007). At the proteome level, hybrids generally show protein levels which deviate from the mid-parent, particularly in functional classes related to central metabolism and stress responses (Marcon et al., 2010). Efforts to map heterosis for various traits generally do not reveal loci for which the association holds universally across genotypes, even for single traits within a species (Huang et al., 2016; Liu et al., 2020). If a particular universal mechanism of heterosis were ultimately revealed, the genes involved would still have biologically additive, dominant, or epistatic gene action. The genes may not be identical at the sequence level, but it would be expected that the mechanism would be common to all cases of heterosis.

## Phenotypic Bases of Heterosis

In hybrid individuals, not all traits are necessarily heterotic (Kaeppler, 2011). Nor is there correlation in levels of heterosis for different traits (Longin et al., 2013; Huang et al., 2015). For example, a hybrid individual might show heterosis in yield and height, but not root angle, and the amount of heterosis for yield and height may differ. The sign of heterosis can vary among traits; inter-subspecific hybrids of *indica* and *japonica* rice (*Oryza sativa*) show increased vigor, but reduced fertility, as do interspecific hybrids of donkeys (*Equus asinus*) and horses (*Equus caballus*; Troyer, 2006; Fu et al., 2014). The degree of heterosis can also depend on environment. Maize hybrids usually show more heterosis in stressful than non-stressful environments, even as overall performance is decreased (Duvick et al., 2004). The lack of consistent levels of heterosis across traits may indicate that heterosis cannot be explained by a unifying, systems-wide mechanism—the reasoning being that all traits would then be affected equally.

Heterosis is also found in complex traits that are a function of multiple component traits, even if the component traits can be fully explained by an additive genetic model. If component traits diverge phenotypically in parents, then heterosis in the complex trait is often detected in progeny even as the component traits remain near the mid-parent (Powers, 1944; Williams, 1959; Grafius, 1961; Coyne, 1965; Melchinger et al., 1994; Dan et al., 2015; Fiévet et al., 2018). For example, in the heterotic pools of oil palm, one pool has a few heavy fruit bunches, and the other has many light fruit bunches (Cros et al., 2015). Their hybrids exhibit substantial heterosis (25%) for fruit production—the product of bunch number and bunch weight—but the hybrid values for bunch number and bunch weight remain near the mid-parent. Notably from the genetics perspective, biological dominance is not required to explain heterosis in multiplicative complex traits (Schnell and Cockerham, 1992; Cros et al., 2015). In this example, it is possible that all of the heterosis in fruit production of oil palms can be fully explained by biologically additive gene action in bunch number and bunch weight (in which case hybrid breeding would not be the optimal strategy to increase fruit production), but in practice the true genetic bases of these traits are unknown.

At the biochemical level, complex phenotypes are a function of multiple component metabolites over time (Fiévet et al., 2018). Metabolite levels or concentrations are themselves a complex phenotype, because they are the product of enzyme amounts and activities within pathways, as well as flux (i.e., rate of turnover) through the pathway (Marshall-Colón et al., 2010; Fiévet et al., 2018). Heterosis can emerge because enzyme activities often affect metabolic flux non-linearly—i.e., halving the activity or concentration of a given enzyme does not necessarily halve metabolic flux (Fiévet et al., 2018; Govindaraju, 2019; Vacher and Small, 2019). The non-linear relationships of enzyme activity and metabolic flux has been proposed as the molecular basis of dominance (Kacser and Burns, 1981). Even if hybrids have enzyme concentrations near the mid-parent, as would be expected under additive inheritance, whether flux or the product metabolite is also at the mid-parent depends on the biochemistry of the pathway (Vacher and Small, 2019). For example, the product metabolite concentration in hybrids may deviate from the mid-parent as enzymes with activities at the mid-parent interact along a pathway and change the flux, or as a rate-limiting step of the pathway is saturated at lower levels than the mid-parent enzyme activity and further increases in enzyme activity do not affect flux (Fiévet et al., 2018). A key conclusion, then, is that non-additive phenotypes such as metabolite concentrations may arise from component additive phenotypes such as enzyme concentrations or activities. Since metabolites are component traits of even more integrated traits, like grain yield, non-additivity in metabolite concentrations can reverberate across levels of phenotype and can lead to heterosis in the integrated trait (Fiévet et al., 2018). Whether heterosis is detected can depend on the choice of phenotype. The metabolome is a phenotype, and using metabolomics data as component traits in multi-trait prediction then has instant appeal, despite current limits in metabolomics on throughput, cost, and the number of metabolites which can be sampled.

## Alternative Definitions of Heterosis

There are several alternative definitions of heterosis which are not equivalent to mid-parent heterosis and do not have a well-defined genetic interpretation. Better parent heterosis (heterobeltiosis) and commercial heterosis, in which either the phenotypic value of the better-performing parent or a commercial check, respectively, is taken from the progeny phenotypic value, may be useful measures for varietal development but have no immediate relevance to genetic improvement of a population by selection, except perhaps to define selection targets (Flint-Garcia et al., 2009; Schnable and Springer, 2013). Better-parent and commercial heterosis might be more informatively described as better-parent and commercial relative performance to avoid equating these measurements with mid-parent heterosis.

Heterosis has also been restricted to describing only increases in progeny vigor relative to parents, i.e., positive heterosis (Shull, 1948). Negative heterosis is observed, as in the progeny of outbreeding depressed parents (Lynch and Walsh, 1998). However, the sign of heterosis can also be a simple artifact of the investigator’s choice of phenotypic measurement (Falconer and Mackay, 1996). For example, positive heterosis for days to flowering is equivalent to negative heterosis for speed of development—a plant which flowers later would have a more positive value for days to flowering, but it would have a less positive value for speed of development since it matures more slowly (Falconer and Mackay, 1996). Therefore, a progeny that flowers later than its mid-parent would show positive heterosis for days to flowering, but negative heterosis for speed of development even though the character measured (when the progeny flowers) is identical. Another common example is that severity of disease can also be viewed as plant health status, and the investigator chooses whether a more positive number represents more or less severe disease symptoms. In the case that a progeny is more resistant to disease that its mid-parent, it will show positive heterosis if less severe disease is measured as a more positive value but negative heterosis if less severe disease is measured as a less positive value.

Finally, heterosis has been conceptualized as a systems-wide phenomenon is which “the increased vigor, size, fruitfulness, speed of development, resistance to disease and to insect pests, or to climactic rigors of any kind” is observed (Shull, 1952). It is perhaps this perspective of heterosis which has fueled the search for a unifying theory of heterosis as well as investigation into its functional genomic causes (Birchler et al., 2003, 2010). Understanding biological bases of heterosis is valuable, but further investigation is needed to use biological insights into heterosis in hybrid crop breeding programs (Ramstein et al., 2019).

## Genomic Prediction

As parents, individuals transmit neither their phenotype nor their full genotype to their offspring. The allele, or more broadly, the gamete is the unit of inheritance. Only the additive portion of genetic value is heritable in the narrow sense if mating is random, because it does not depend on intra- or inter-locus combinations of loci which are potentially disrupted upon mating (Falconer and Mackay, 1996). If mating is random, additive genetic value is all that is maximizable or “breedable” cyclically over generations, and an individual’s additive genetic value is its breeding value (Falconer and Mackay, 1996; Huang and Mackay, 2016).

Despite its name, the concept of breeding value was not developed specifically for the purpose of breeding, but rather to explain the inheritance of quantitative traits: because Mendel discovered inheritance in traits which had discrete classes, it was initially unknown whether continuous, quantitative traits were also controlled by genes that could be transmitted from parent to progeny (Bernardo, 2020). Fisher (1918) not only conceptualized that quantitative traits could be the effect of many genes, but also connected the partial inheritance of parental alleles to observed patterns of resemblances among relatives (Bernardo, 2020). In applied breeding programs, some of the assumptions that define breeding value—such as random mating—are routinely unmet (Falconer, 1985; Bernardo, 2020). Recently, it has been suggested to move away from referring to estimates of transmissible variance as breeding values in applied plant breeding programs for clarity and because non-additive variance can be transmitted via cross selection (Bernardo, 2020; Werner et al., 2020). Here, we refer to breeding values even though at times the definition is not strictly met.

True breeding value cannot be measured or even observed in the individual alone, since true measure of breeding value requires errorless observations of every possible progeny resulting from the individual mated to every possible member of the population to which it belongs. Therefore, breeding values are estimated. The estimation of breeding values was first accomplished by progeny testing. With random mating, the average performance of an individual’s progeny is an estimate of its breeding value (Supplementary Table 1; Eq. 17). Many mating schemes were developed to more accurately estimate breeding values by use of more types of relatives (Hallauer et al., 2010). However, best linear unbiased prediction (BLUP) was developed to estimate breeding values without the need for mating designs, as pedigree-based variance-covariance relationship matrices describe the resemblance between relatives in a linear mixed model (Supplementary Table 1; Eq. 18; Henderson, 1975). Assuming no fixed effects, BLUP of breeding value can be thought of as a linear combination of observed phenotypes weighted by the degree of their relationship with the individual for which breeding value is predicted. The pedigree-based relationship matrix is often referred to as the numerator relationship matrix, A, and pedigree-based BLUP is sometimes called ABLUP (Bernardo, 2002; Gianola et al., 2018). Interestingly, BLUP was slow to gain traction in plant breeding, but quickly became popular in animal breeding due to the standing practical impossibility of replicating animal genotypes (Piepho et al., 2008). It was perhaps here that the two fields decoupled in their study of genomic prediction and selection, and the benefits of cross-disciplinary synchronization of methods are recognized in both fields (Schön and Simianer, 2015; Hickey et al., 2017).

Well in advance of the sequencing technologies that would make markers cheaper, less biased, and more representative of the genome, the framework for genomic prediction of EBV using molecular markers was developed. Bernardo (1994; 1996) used genome-wide markers to estimate breeding values from kinship rather than pedigree in the first instance of genomic prediction. Whittaker et al. (2000) addressed the problem of selection of marker subsets for linear regression in marker-assisted selection (MAS) by ridge regression, which is a regularization method that shrinks normalized effects for all markers equally toward an assumed mean of zero by an optimized parameter, λ (Supplementary Table 1; Eq. 19). This was a crucial advance for the use of genomic markers in selection, because markers generally outnumber phenotypes and cause the “large p, small n” problem: linear regression by OLS is not possible if predictors (p) outnumber responses (n), and subsampling is usually suboptimal (Whittaker et al., 2000). Ridge regression, like other regularization methods, addresses the problem of selecting predictors by shrinking their coefficients instead of subsampling. Regularization also reduces model overfitting, in which models capture noise (i.e., residual error) as well as signal (i.e., effects of predictors). Both model overfitting and poor choice of predictors reduce prediction accuracies. Meuwissen et al. (2001) realized that if markers in linkage with every quantitative trait locus (QTL) affecting a trait were to become available, then additive effects per marker (estimated by ridge regression or other methods) could be summed to calculate individuals’ genomic estimated breeding values (GEBVs). Use of GEBVs or any other value estimated using genome-wide information for selection is referred to as genomic selection.

Since 2001, tens of methods for genomic prediction of breeding values, as well as the genetic values of lines used for production rather than breeding, have been developed (Gianola et al., 2006, 2009; Legarra et al., 2009; Habier et al., 2011; Momen et al., 2018; Wang et al., 2018; Howard et al., 2019; Kadam and Lorenz, 2019). These methods include both frequentist and Bayesian, as well as parametric and non-parametric, methods (Gianola et al., 2018). The parametric method of Whittaker et al. (2000), ridge regression of marker effects, is called RR-BLUP and assumes marker effects are drawn from a normal distribution. Hayes et al. (2009) later showed that estimation of breeding values by RR-BLUP is equivalent to estimation by genomic BLUP (GBLUP), in which markers are used to compute a genomic relationship matrix. The genomic relationship matrix (often denoted G) replaces the pedigree relationship matrix (A) to calculate BLUPs of GEBVs (VanRaden, 2008). GBLUP is generally more accurate than ABLUP, because realized genetic relationships deviate from pedigree expectations following Mendelian sampling, selection, and other events (VanRaden, 2008). RR-BLUP and GBLUP are widely used for genomic prediction because they are relatively straightforward to interpret, often more computationally efficient, and often as accurate as other methods with more realistic assumptions (Zhao et al., 2015b; Howard et al., 2019). For reviews of genomic prediction methods, see Gianola et al. (2018) and Howard et al. (2019).

In hybrid breeding, genomic prediction can be used to (a) predict the combining abilities of inbred lines, and (b) predict the performance of new hybrid genotypes (Bernardo and Yu, 2007; Technow et al., 2012). To predict the combining abilities of inbred lines, phenotypes of their hybrid progeny are used to estimate inbred combining abilities, then the combining abilities of the inbred lines are modeled as a function of their inbred genotypes (Bernardo and Yu, 2007). To predict the performance of new hybrid genotypes, hybrid phenotypes are modeled as a function of hybrid genotypes (Technow et al., 2012). However, hybrid genotypes are usually not sequenced directly, and are inferred instead by genotyping their inbred parents, which reduces the total number of individuals for genotyping. Even though within hybrid genotypes a given allele may be specific to a particular population, modeling population-specific effects of alleles has not been shown to greatly increase accuracy in predicting hybrid performance (Technow et al., 2012).

Prediction accuracy is an important determinant of whether genomic prediction will lead to effective selection across environments, years, and genotypes. Factors which influence accuracy of GEBVs include choice of statistical model, trait heritability, precision in geno- and phenotyping, size the of the training set, and relatedness/common LD structure of the training and testing set (Heslot et al., 2015; Rutkoski et al., 2017). Modeling non-additive effects is an active area of research with particular relevance to prediction of GEBV or performance (Vitezica et al., 2017; Varona et al., 2018b; Voss-Fels et al., 2019). Dominance deviations are by definition zero in genetic values of homozygous inbred lines; only additive and epistatic additive effects are non-zero. In non-inbred individuals, all non-additive effects contribute to genetic value. Product development, in contrast to population improvement, is concerned with total genetic value, which includes non-additive effects.

Though modeling non-additive effects would be expected, then, to improve prediction accuracies, a reminder is warranted: modeling non-additive genetic effects will only improve prediction accuracies if non-additive genetic effects exist for the traits of interest and non-additive genetic effects can be estimated accurately in the populations of interest (Hill et al., 2008). In light of these considerations, it is perhaps unsurprising that in practice classical models which fit non-additive effects rarely outperform accuracies of additive models (Varona et al., 2018a; Werner et al., 2018). Interestingly, though, if dominance effects are fit in absence of underlying dominance, Duenk et al. (2017) observed no change in accuracy of estimating additive effects. In fact, accuracy of estimation of additive effects was always improved or unchanged by models which incorporated dominance, even in small sample sizes and/or in cases that genetic variance explained low proportions of phenotypic variance (Duenk et al., 2017). Though no similar study has been conducted for epistasis to our knowledge, there appears to be no penalty to fitting dominance effects. In crossbred (hybrid) and pure-line animals, incorporating positive directional dominance effects and inbreeding depression effects (which are posited to underlie heterosis) sometimes improves prediction accuracies relative to assuming dominance effects centered at zero or ignoring inbreeding (Xiang et al., 2016; Varona et al., 2018a; Christensen et al., 2019). Inclusion of non-additive effects can also improve choice of parents for crossing by estimates of their progeny genetic value (Aliloo et al., 2017; Werner et al., 2020).

Multivariate genomic prediction methods are promising for improving prediction accuracy when traits under selection with low heritability are genetically correlated with traits with high heritability (Jia and Jannink, 2012; Neyhart et al., 2017; Okeke et al., 2017; Sun et al., 2017; Wang et al., 2017; Fernandes et al., 2018; Watson et al., 2019). Because heterosis in complex traits can sometimes be explained by component traits which are negatively complementary in the parents, multivariate genomic prediction could potentially improve predictions of hybrid performance and EBVs if such component traits are included. Hybrid production also faces a constraint on the performance of the inbred parents, in that inbred parents must have good *per se* performance and specific male and female morphotypes for hybrid seed production (Hallauer et al., 2010). Generally, inbred parents are selected for these traits separately from their selection as hybrid parents (Hallauer et al., 2010). Treating inbred and hybrid performance as different but genetically correlated traits in multivariate genomic selection may improve selection accuracy for hybrid performance, but this has not been reported to date.

## Genomic Selection in Hybrid Breeding

**FIGURE 2:**
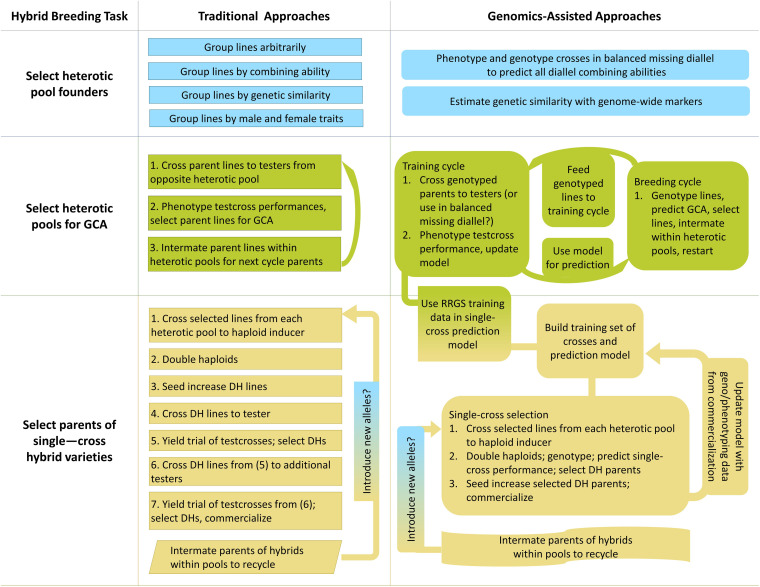
Graphical overview of traditional and genomics-assisted hybrid crop breeding pipelines.

Breeding for hybrid performance can benefit from the incorporation of genomic selection, and in a few cases genomic prediction could be used to develop new breeding strategies (Xu et al., 2017). Hybrid breeding primarily involves inter-population improvement, in which recurrent selection of individuals within populations is effected between populations by selecting on individuals’ performance as parents in between-population crosses (Hallauer et al., 2010). Unlike intra-population improvement, in which performance of crosses within populations is used to recurrently select individuals in the same population, inter-population recurrent improvement is not only of the populations themselves but also of the performance of their hybrid crosses in combination (Hallauer et al., 2010). Hybrid breeding can be considered to have three main modules: selecting founders of heterotic pools, breeding heterotic pools, and selecting parents of crosses for production pipelines (Figure 2).

Heterotic pools are distinct groups of lines which reliably produce heterosis upon crossing; the lines may or may not be related (Melchinger and Gumber, 1998). Breeding distinct heterotic pools is more effective in consistently producing high-performing hybrids than making random crosses, because heterotic pools are improved by recurrent selection for average line performance in hybrid crosses with the opposite heterotic pool, which is termed general combining ability (GCA; Sprague and Tatum, 1942; Reif et al., 2005). Hybrid performance is modeled as the sum of each parental GCA and the specific combining ability, or SCA, of the parent pair (Supplementary Table 1; Eq. 20; Griffing, 1956b). GCA corresponds to additive effects, whereas SCA corresponds to dominance effects (Griffing, 1956a). The process of breeding heterotic pools increases the ratio of GCA to SCA effects over time, so the parents’ performance in crosses becomes more heritable in the narrow sense (Schulthess et al., 2017). In addition, distinguishing heterotic pools addresses the practical need for lines to have specific traits for use as males or females, as male and female traits such as high pollen production or male sterility can be pool-specific (Zhao et al., 2015b).

Heterotic pools are developed by choosing founders, then recurrently improving the pools for combining ability. Historically, hybrid breeding was first systematically conducted in maize in North America, and maize breeding programs are the longest-running among hybrid crops (Shull, 1908). Though it is a common misconception that the founders of the first heterotic pools of maize were chosen for their origin in geographically and genetically distinct groups, in fact the archetypal Reid-Lancaster heterotic pattern was developed empirically by trial-and-error in crossing (Melchinger and Gumber, 1998; Tracy and Chandler, 2006). Later, successful commercial maize heterotic pools arose upon separating of lines into groups for use as males or females; the initial pools used have been posited to have shared around half of their genetic background (Tracy and Chandler, 2006). Observed genetic divergence between the first North American maize heterotic pools developed in response to selection and drift during breeding, rather than by selection of divergent founders (Duvick et al., 2004; van Heerwaarden et al., 2012).

Heterotic pools have not been widely established for major crops such as wheat and rice, and there is interest in methods to choose founders of heterotic pools (Wang et al., 2015; Zhao et al., 2015a). Based on evidence from maize, some authors suggest that any split of available germplasm will allow development of heterotic pools by breeding, and founder grouping is relatively unimportant (Cress, 1966; Lee and Tracy, 2009). Others suggest systematic approaches. To choose founders of heterotic pools, Melchinger and Gumber (1998) proposed to form genetically similar groups of individuals, then cross a manageable number of representatives of each divergent genetic subgroup and test their progeny performance in replicated field trials. Founders can then be chosen for high *per se* performance, high average progeny performance and progeny genetic variance, and—as applicable—suitability for use as males or females (Melchinger and Gumber, 1998; Melchinger, 1999). Use of genetically diverged founders increases the ratio of GCA to SCA, and heterosis due to dominance is expected to be positively correlated with increasing genetic distance of parents (Falconer and Mackay, 1996; Reif et al., 2007). In practice, positive heterosis is often observed with increasing genetic distance until a point, at which outbreeding depression prevails and heterosis is negative (East, 1936). A simulation study of heterotic pool formation in an autogamous crop compared randomly splitting the founder population, splitting the founder population by genetic distance, and optimizing the founder split by performance of their F_1_ hybrids, and found no differences in future hybrid performance among the formation strategies, suggesting that the initial split can be arbitrary (Cowling et al., 2020). However, no population structure in the founder population was assumed, and testing the formation strategies given a structured founder population would be of interest. Even though existing heterotic pools of maize, for example, were not established from genetically distinct lines, testing the optimal strategy to form heterotic pools in an allogamous species would be interesting as well (Duvick et al., 2004).

Genomics-assisted approaches to choose founders have been proposed (Zhao et al., 2015a; Boeven et al., 2016). One approach is to simply extend the aforementioned method by using genome-wide markers to identify genetic subgroups (Boeven et al., 2016). Another approach to select founders of heterotic pools is to use a training set of observed hybrid crosses of founders to predict performance of unobserved crosses (Zhao et al., 2015a). Then, groups of lines which are heterotic in combination can be identified algorithmically for selection (Zhao et al., 2015a). The long-term potential of the groups of lines can be further assessed by simulation for their genetic representativeness of the base population, usefulness (in terms of the initial population mean and expected response to selection), and long-term selection limits (Zhao et al., 2015a). Though this approach has not been empirically validated, it has been initialized in rice and wheat (Zhao et al., 2015a; Beukert et al., 2017). In the wheat population surveyed, only sixteen of the 135 individuals surveyed were needed to maximize usefulness and the long-term selection limit (Zhao et al., 2015a). Therefore, if effective, this approach could dramatically reduce resources needed to screen potential founders of heterotic pools. It would be interesting to test whether this particular method would discern the founders of North American maize heterotic pools as optimal or near-optimal.

Once founders have been chosen, heterotic pools must be developed by breeding. Heterotic pools can be recurrently improved for their ability to combine into hybrids with high performance by reciprocal recurrent selection (RRS; Comstock et al., 1949). In the first generation, lines from each heterotic pool are at once selfed and crossed to the opposite heterotic pool (Comstock et al., 1949). Rather than making every line-by-line cross, one or more random testers are chosen to represent each heterotic pool and used for crossing to all lines of the opposite heterotic pool, hereafter referred to as testcrossing (Comstock et al., 1949). In the next season, the testcrosses are grown and the testcross phenotypes are used to determine GCA of their parents (Comstock et al., 1949). Parents are selected for their GCA, and in the final season of the cycle, the selected parents are grown from saved seed and randomly intermated within heterotic pools (Comstock et al., 1949). The cycle begins again, with no need to inbreed the parents (Comstock et al., 1949). Overall, RRS can be thought of as a special case of standard phenotypic selection, where the phenotype in question is combining ability, and measuring combining ability requires progeny testing.

Reciprocal recurrent selection, then, is an ideal candidate for genomic selection. Phenotyping GCA is expensive and time-consuming. If GCA could be predicted in the first generation of the RRS cycle, then the selected lines could be intermated immediately, reducing cycle time by two-thirds. Reciprocal recurrent genomic selection (RRGS) has been studied by simulation in oil palm (Ibáñz-Escriche et al., 2009; Kinghorn et al., 2010; Cros et al., 2015). Cros et al. (2015) investigated the effects of training set composition, frequency of model calibration by progeny test, and number of selection candidates on annual selection response. If number of available selection candidates was controlled, RRGS showed a 48% advantage in annual selection response over RRS because genomic predictions replaced phenotyping by progeny testing. If RRGS was assumed to permit evaluation of twice as many candidates for selection relative to RRS as progeny testing was reduced, then the advantage increased to 72%. Interestingly, Cros et al. (2015) tested whether including hybrid geno- and phenotypes in the training set improved accuracy more than including the parents alone and found accuracy to be sensitive to the frequency of model calibration and the number of hybrids included. They posited that optimal number of F_1_ hybrid genotypes in the training set should increase with heterozygosity of the parents of the F_1_ hybrids, because more heterozygous parents may produce more within-cross variance than less heterozygous parents (Cros et al., 2015). In a follow-up study, Cros et al. (2018) also investigated whether prediction accuracies from a training set of genotypes from either only the previous breeding cycle, or both the previous two breeding cycles, was superior in RRGS. They found that training on two previous breeding cycles was superior because of both increased in prediction accuracy and slightly decreased loss of additive genetic variance (Cros et al., 2018).

A key consideration in both studies was that dominance was not simulated even though RRGS was used, so the mean genetic values of the hybrids were equal to the mean of their parents (Cros et al., 2015, 2018). Further simulations of RRGS with dominance would be valuable. If dominance were simulated, then the F_1_ hybrids’ mean genetic value would differ from the parents’ mean genetic value. To use both parent and hybrid geno- and phenotypes in the same training model when directional dominance is present, it would likely be necessary to include a fixed effect for the average heterozygosity of each individuals’ genotype following Xiang et al. (2016) and Vitezica et al. (2016), or alternately to estimate hybrid and parent BLUPs of phenotype separately following Liang et al. (2018), in order to accurately predict parental GCA or hybrid genetic value.

Reciprocal recurrent selection differs slightly from recurrent selection within populations in that breeding values depend on allele frequencies in both heterotic pools (Stuber and Cockerham, 1966). Two issues then arise. Rembe et al. (2019) note that in its current implementation, RRGS does not optimize frequencies of overdominant alleles (either positive or negative) and in some cases of negative overdominance will fix unfavorable alleles. RRGS also cannot optimize frequencies of alleles which have a frequency of zero in either population, nor predict their effects, and maximum genetic potential cannot then be achieved (Cress, 1966; Kinghorn et al., 2010). Periodic introduction of new germplasm to refresh heterotic pools might overcome the latter issue if it is in fact significant, though care must be taken not to disrupt the heterotic pattern. Another interesting but unexplored possibility is to avoid unwanted fixation from the start of RRGS; methods developed to control long-term inbreeding under genomic selection might be adaptable for the latter purpose.

As heterotic pools are developed, the next module is initiated: parents of crosses for production pipelines are selected. First, individuals are selected from each heterotic pool (Lee and Tracy, 2009). In established commercial maize hybrid breeding programs, within-pool lines which have a past record of producing high-performing cross-pool hybrids are recycled by crossing, and it is their progeny which are selected (Mikel and Dudley, 2006). As of 2006, only seven inbred founder lines (though from four heterotic pools) were thought to be the origin of the commercial North American breeding pool (Mikel and Dudley, 2006).

Next, selected individuals from each pool are used to develop inbred lines by doubled haploid (DH) production or selfing. During inbreeding of lines from two-parent crosses or upon availability of DH lines, lines are selected for *per se* performance, often for traits such as disease resistance (Lee and Tracy, 2009; Kadam and Lorenz, 2018). Then, the selected lines are testcrossed, usually to a single tester, and selected by the performance of their hybrids in a few environments (Lee and Tracy, 2009). Only these selected lines are crossed again (after selfing to homozygosity if DH lines are not used) to multiple testers, and their hybrids are advanced to multi-environment trials (METs; Lee and Tracy, 2009). Parental inbred lines which produce outstanding hybrids can then be commercialized, and their hybrids may be used in production (Lee and Tracy, 2009).

Some authors have proposed to reduce or eliminate preliminary testing by use of genomic prediction (Lee and Tracy, 2009; Kadam et al., 2016). With sufficient prediction accuracies, genomic predictions of all possible two-parent, single crosses of a set of inbred lines (i.e., a diallel) could replace testcrossing, then crosses predicted to have outstanding performance could immediately be tested in METs (Hallauer et al., 2010; Kadam et al., 2016). Genomic prediction could save time and resources as well as retaining useful lines which happen to perform poorly with chosen testers (Kadam et al., 2016). The primary challenges in doing so are generating an adequate training set of crosses and predicting SCA; it remains difficult to predict the performance of hybrids for which neither parent is observed in the training set (Kadam et al., 2016; Kadam and Lorenz, 2019). Notably, the ideal training set to predict performance of single crosses that would be obtained from a diallel is thought to be not a set of testcrosses, but rather the North Carolina II (NCII) design, in which inbred parents are grouped into males and females, then crossed factorially across groups (Hallauer et al., 2010; Fristche-Neto et al., 2018). However, the number of crosses needed for training can be reduced from NCII by using various algorithms which rely on estimates of relationship (Fristche-Neto et al., 2018; Akdemir and Isidro-Sánchez, 2019; Guo et al., 2019). Inclusion of historical single cross information can also improve prediction accuracies, though in some studies this benefit was only realized if the crosses were from recent cycles, even within the same breeding program (Dias et al., 2019; Schrag et al., 2019). If the production of F_1_ hybrid seed by cross-pollination is too expensive on a large scale with many hybrid combinations (as in self-pollinated crops), then F_1:2_ individuals can be substituted into the training set with only modest reductions in prediction accuracies (Technow, 2019). If entirely eliminating testcrossing is perceived as too risky, selections from testcrossing can be supplemented with predicted exceptional single crosses (Kadam and Lorenz, 2018; Viana et al., 2018). Another cost-reducing alternative is to testcross a subset of several related lines, and predict combining abilities for their relatives (Windhausen et al., 2012). Once exceptional single crosses are identified, with or without testcrossing, their seed can be increased, advanced through preliminary and multi-environment yield trials, and eventually released as varieties for production (Kadam and Lorenz, 2018).

Other approaches to utilizing heterosis, besides by inbred development and testing, deserve consideration. The cost and time required for traditional and genomic hybrid breeding is substantial, and thus the rate of genetic gain is generally less for hybrid than inbred breeding (Longin et al., 2012). Furthermore, hybrid seed production is generally more expensive that inbred seed production, and hybrid genotypes cannot be replicated by selfing (Schulthess et al., 2017).

One alternative approach to using heterosis is to systematically reproduce desirable non-inbred genotypes. A major barrier to utilization of superior genotypes in non-inbred populations is that they cannot be repeatedly reproduced identically by crossing, since their parents are not fully inbred (Wricke and Weber, 1986). However, recent proof-of-concept “reverse breeding” in *Arabidopsis thaliana* offers an alternative to fixing heterosis by crossing inbred lines (Wijnker et al., 2012). In reverse breeding, recombination is suppressed in non-inbred lines, and DH lines are generated from their gametes (Wijnker et al., 2012). The DH lines can then be maintained, genotyped, and crossed at will to reconstitute the original non-inbred line (Wijnker et al., 2012). However, this method has not been tested in crop species or applied in crop breeding. A similar approach is synthetic apomixis, in which seeds identical to the parent plant are produced without meiosis or fertilization (Wang et al., 2019). In rice, apomictic seeds can be produced by editing only four genes, but fertility issues leading to low seed set also result (Wang et al., 2019).

Clonal propagation methods also reproduce non-inbred genotypes. Many non-inbred economically important crops, including sugarcane, potato, and cassava, are propagated asexually as clones rather than from seed (McKey et al., 2010). The drawbacks of clonal propagation, however, include the accumulation of deleterious somatic mutations, disease, costs of propagule production, and the recalcitrance of some species to clonally propagate (McKey et al., 2010). Use of polyploidy has also been viewed as a way to “immortalize” hybrids, as allopolyploids can maintain heterozygosity across their subgenomes at individual loci even upon selfing (Santantonio et al., 2019). Dosage effects, or changes in phenotype due to increases in allele copy number, independent of allele state, have also been posited to contribute to the genetic values of polyploids relative to genotypes of lesser ploidies (Gianinetti, 2013; Yao et al., 2013; Fort et al., 2016). Unlike in diploids, heterosis in polyploids is not maximized in a single cross; this phenomenon is termed progressive heterosis (Washburn and Birchler, 2014). Progressive heterosis is expected in polyploids because the number of gametes inherited exceeds the number of parents. Going beyond single crosses permits combining gametes from more than two parents into a single individual genotype, so additional heterosis results. For example, in autotetraploids, heterosis would be maximized by a four-way cross. In diploids, the number of gametes inherited by the F_1_ progeny in a single cross (two) is equal to the number of parents (two), so heterosis is maximized in single crosses.

If a uniform population is not necessary, then breeding open-pollinated varieties (OPVs) can lead to effective utilization of heterosis. For much of human history, OPVs were the only varieties available. This is still true in regions which the commercial breeding sector does not yet serve, and OPVs can outperform hybrids in some low-input environments (Pixley, 2006; Masuka et al., 2017; Andorf et al., 2019). In United States maize production, OPVs were abandoned in the early 1920s due to the difficulty of improving their quantitative traits (i.e., yield) as well as lack of uniformity (Duvick, 1999). However, it is unknown whether OPVs could outperform hybrids today if they had been as intensively developed (Duvick, 1999). OPV breeding could be advanced by genomic selection; the breeding cycle for OPVs is shorter and less costly than for hybrids. Furthermore, if used as part of a reverse breeding pipeline, sufficiently outstanding individuals from OPVs could be reproduced indefinitely as uniform “hybrid” varieties.

If heterosis is largely due to dominance rather than overdominance, then inbred lines which perform as well as hybrids must be possible, although they may take time to develop due to linkage disequilibrium (Werner et al., 2020). There is some evidence from commercial maize programs that inbred lines bred conventionally are already beginning to approach hybrid line performance, though likely because of the longer hybrid breeding cycle (Troyer and Wellin, 2009). Heterotic effects of yield have decreased as a percentage of mean yield over a short time—100 years—perhaps also because some favorable dominant alleles have been fixed in inbreds. Continued purging of deleterious recessive alleles from the genome by genome editing has been proposed, especially in regions hard to reach by recombination such as the centromere (Wallace et al., 2018; Valluru et al., 2019). If overdominance also affects hybrid performance, and overdominant loci can be identified, then arguably copy number variation could be induced to fix overdominance in inbred lines. These genomics-assisted approaches are reminiscent of genetic ideotype building, but until they are possible, genomics-assisted inbred line breeding may be a good start to genomics-assisted ideotype building of inbred lines (Trethowan, 2014).

## Long-Term Optimization of Selection in Genomic Selection Programs

All plant breeding programs require genetic variance for continued progress. Within any breeding population, reducing effective population size by selection early in the program may limit long-term genetic gain (Comstock et al., 1949; Robertson, 1960; Woolliams et al., 2015). Though genomic selection leads to less inbreeding than pedigree-based selection methods, inbreeding must be controlled (Rodríguez-Ramilo et al., 2015; Woolliams et al., 2015). Direct selection on GEBV maximizes gain in the subsequent cycle only and does not necessarily maximize long-term gain (Sonesson et al., 2012). Fortunately, data collected routinely in genomic selection programs allow monitoring and optimization of loss of diversity and inbreeding. Genomic selection strategies which seek to balance rates of genetic gain and loss of diversity include:

(a) Optimum contribution selection: genetic value is maximized while inbreeding is constrained to give the optimal contributions of parents to the next generation, i.e., number of progeny (Meuwissen and Sonesson, 1998).
(b) Weighting of rare alleles: allelic effects are weighted by their frequency such that rare favorable alleles are preserved (Goddard, 2009).
(c) Weighted genomic selection: allelic effects are weighted by their frequency, but also the magnitude of their effect, such that rare favorable alleles which tend to have large effects on EBV are preserved (Jannink, 2010).
(d) Genotype building: a subpopulation is selected algorithmically to segregate for maximal haplotype values, then intermated such that the two best segments ultimately segregate with equal frequency (Kemper et al., 2012).
(e) Optimal cross selection: selection intensity, inbreeding, and cross allocation are simultaneously optimized (Gorjanc et al., 2018).
(f) Usefulness criterion parental contribution: overall and within-family selection intensity, inbreeding, and cross allocation are simultaneously optimized (Allier et al., 2019).
(g) Genomic mating: genetic value, inbreeding, and risk (calculated from variability in breeding value estimates) are simultaneously optimized (Akdemir and Sánchez, 2016).
(h) Optimal haploid value selection: outbred individuals are selected for the predicted value of the best DH lines they could produce, then used to make DH lines for which breeding or genetic values are then predicted (Daetwyler et al., 2015).
(i) Optimal population value selection: sets of individuals are selected for their collective rather than individual maximum possible haploid value (Goiffon et al., 2017).
(j) Expected maximum haploid breeding value selection: individuals are selected for their maximum possible haploid value (Müller et al., 2018).
(k) IND-HE: genetic gain and expected heterozygosity are balanced in selection (De Beukelaer et al., 2017).
(l) Look-ahead selection: sets of individuals are selected for their collective rather than individual maximum possible haploid value, with the maximum value occurring in a user-specified target generation (Moeinizade et al., 2019).
(m) Optimal contribution selection to update the reference population: assuming a breeding population is used to update the training set for prediction, selecting the training set candidates by optimal contribution selection balances genetic gain and inbreeding (Eynard et al., 2018).
(n) Optimal contribution selection with branching: the population mating scheme is branched into two paths which maintain genetic diversity and maximize genetic gain (Santantonio and Robbins, 2020).

A simulation comparing all methods of long-term selection optimization in hybrid breeding programs is not yet available (Rembe et al., 2019). Development of genomic selection strategies to optimally introgress novel variation are also ongoing (Rembe et al., 2019). A recent comparison of introducing genetic donors with varying performance levels either using or omitting a bridging population to increase mean genetic values of introgression lines found that use of a bridging step was more useful when considering low-value donors, and that controlled introduction of diversity increased gain relative to a completely closed population (Allier et al., 2020a). Though the field of long-term selection optimization developed in response to need to avoid inbreeding and maintain genetic variance, the techniques developed can also be used to improve short- and medium-term gain (Müller et al., 2018).

For hybrid programs specifically, selection optimization methods to prevent unintentional allelic fixation during RRGS in opposite heterotic groups could be useful (Cowling et al., 2020). Another issue in hybrid breeding over time is introducing new germplasm and assigning it to a heterotic pool. Traditionally, new individuals are assigned to a heterotic pool by their phenotypic similarity to existing members or observed performance in testcrosses with representatives of each pool (Melchinger, 1999). Alternatively, individuals can be assigned to pools by genetic resemblance (Melchinger, 1999; Boeven et al., 2016). However, in practice, genetic distance is not consistently useful in assigning individuals to heterotic pools (Fischer et al., 2010; Brauner et al., 2019).

Though advanced commercial maize hybrid breeding programs should not be construed as resulting from long-term genomic selection, the genetic base of North American and European commercial maize is narrow, prompting concern that limiting loss of diversity has occurred (Brauner et al., 2019; Allier et al., 2020b). Some approaches, such as Germplasm Enhancement of Maize (GEM), have proposed adaptation of exotic germplasm to commercial inbred backgrounds by public-private collaboration. Several inbred lines have been released as a result of GEM (Samayoa et al., 2018). Other efforts based on generating DH lines of maize landraces and characterizing them for their *per se* and testcross performance with European testers have also demonstrated a 15% yield gap between mean testcross yield and mean commercial yield (Brauner et al., 2019; Hölker et al., 2019). Genomic selection for line adaptation has been proposed but is largely untested (Bernardo, 2009; Samayoa et al., 2018; Allier et al., 2020b). Additionally, commercial breeding programs may reduce loss of useful diversity by targeted introgression of QTL or transgenes into elite lines, which improves the lines without drastically changing their genomic makeup or disrupting the heterotic pattern (Samayoa et al., 2018).

The limits of long-term selection within closed breeding populations are unknown (Dudley and Lambert, 2004; Paixão and Barton, 2016). Breeding progress for high grain oil and protein content, which was initiated in a maize OPV, has continued for over 100 cycles of selection without introduction of new germplasm (Dudley and Lambert, 2004; Moose et al., 2004). In the same experiment, breeding progress for low oil and protein content ceased due to measurement and physiological constraints, respectively (Dudley and Lambert, 2004). Surprisingly, when the direction of selection on lines bred for low oil and protein content was experimentally reversed at 48 generations, selection response in the opposite direction occurred rapidly (Dudley and Lambert, 2004). Though not conclusive, these results suggest that it is difficult to exhaust response to selection even in completely closed or selected populations using conventional recurrent selection strategies. The cost of testing and adapting vast quantities of new germplasm may not be worth the short-term benefits for advanced commercial hybrid programs if sufficient genetic variance remains for selection gain, even among very few lines.

## Discussion

Exact recommendations for crop hybrid breeding programs are situation-dependent, including whether and how to apply genomic selection. Factors to consider in implementation of genomic selection strategies include budget, trait heritability, cost and accuracy of phenotyping, length of the breeding cycle, and infrastructure for genomic selection (e.g., marker availability, marker cost, bioinformatics software, statistical expertise, etc.; Heslot et al., 2015). General factors that affect the success of a hybrid breeding program relative to an inbred breeding program include (a) mating system, including whether selfing is possible, (b) existence of heterotic pools, (c) the degree of heterosis, (d) the cost of hybrid seed production, including availability of hybridization systems, and (e) the number of seeds needed in the cropping system (Longin et al., 2013). Another rationale for hybrid breeding has been that the sale of hybrid seeds generates a sustainable funding model for breeding, with built-in variety protection (Schulthess et al., 2017). However, this argument is beyond the scope of plant breeding and requires economics research.

Mating system is a major factor driving the use of hybrid breeding systems (Longin et al., 2013). Most hybrid crops (e.g., maize, sugarbeet, rye, and sunflower) are allogamous, or outcrossing, rather than autogamous, or selfing. Though both present difficulties in breeding programs—autogamous crops may be difficult to cross, and allogamous crops may be difficult or nearly impossible to self—in general autogamous crops are less amenable to hybrid breeding due to higher costs of seed production and less observed heterosis (Wricke and Weber, 1986; Longin et al., 2012). Less heterosis in autogamous than allogamous crops may have an evolutionary basis. Over time, deleterious recessive mutations are more exposed to selection in selfing than outcrossing species—ultimately leading to reduced inbreeding depression (Moose et al., 2004). Selfing genotypes also have more opportunities for selection on epistatic networks, perhaps leading to increased outbreeding depression (Fenster et al., 1997).

For a breeding program, the question then remains whether the gains of heterosis are outweighed by the costs of breeding hybrids in autogamous crops. The costs of breeding hybrids can be reduced by developing male-female heterotic pools, scalable male sterility systems, and hybridization systems. The gains of heterosis can be increased by breeding heterotic pools. Thus, initial investment to establish a hybrid breeding program may be high, but it could provide higher returns over time than an inbred program. A case study of hybrid wheat, for example, found that although hybrids are currently competitive with inbred varieties, whether long-term improvement of hybrids keeps pace with lines strongly depends on budget, cost of hybrid seed production, and GCA variance (Longin et al., 2014). Use of genomic prediction can increase the relative efficiency of hybrid breeding to line breeding (Longin et al., 2015). If sufficient budgets to cover the start-up costs of hybrid breeding (e.g., heterotic pool development, male sterility systems) are available at no cost to line breeding, then hybrid breeding is worth investigating.

Whether for autogamous or allogamous species, genomic selection methods have potential to increase rates of genetic gain at every stage of hybrid breeding. Use of genomic selection, for example, to rapidly develop heterotic pools in crops in which they are not well-established—e.g., rice and wheat— is worth trying (Rembe et al., 2019). Further reports on RRGS programs which have been initiated in oil palm, which has a long generation interval, high phenotyping costs, and high environmental impact, are anticipated (Cros et al., 2017; Nyouma et al., 2019). In selection of single crosses, genomic prediction has potential to reduce the need for testcrossing and field evaluation (Longin et al., 2015; Kadam et al., 2016). Longin et al. (2015) considered optimal allocation of resources to number of DH lines, test locations, and tester lines used for inbred and hybrid breeding programs with different degrees of reliance on genomic prediction and different prediction accuracies. After DH production, testcrosses or lines were either immediately subject to genomic selection, advanced through one round of field testing, or advanced through two rounds of field testing (Longin et al., 2015). The importance of field testing strongly depended on accuracies of genomic predictions, but for hybrid breeding, even prediction with low accuracies improved rate of genetic gain (Longin et al., 2015). If DH lines underwent a round of phenotypic selection before advancing, the relative merits of incorporating genomic selection did not change (Marulanda et al., 2016). The best scenario was genomic prediction followed by a round of phenotyping (Longin et al., 2015; Marulanda et al., 2016).

The era of genomic selection offers new opportunities in hybrid breeding. Genomic selection methods can jumpstart the establishment of heterotic pools by founder selection and use of RRGS to unlock heterosis in new hybrid breeding programs. Genomic selection can also shorten the notoriously long hybrid breeding cycle by reducing the need for testcrosses and their phenotypic evaluation. Though implementing genomic selection methods requires optimization to specific hybrid breeding situations, a sufficient framework for breeders to make genomics-assisted decisions already exists.

## Author Contributions

ML conducted the literature review and wrote the manuscript. AS and JR conceived of the review topic, edited the manuscript, and generated cross-disciplinary insights through discussion. All authors contributed to the article and approved the submitted version.

## Conflict of Interest

The authors declare that the research was conducted in the absence of any commercial or financial relationships that could be construed as a potential conflict of interest.

## References

[B1] Akdemir D. Isidro-Sánchez J. (2019). Design of training populations for selective phenotyping in genomic prediction. *Sci. Rep.* 9:1146. 10.1038/s41598-018-38081-6 PMC6363789 30723226

[B2] Akdemir D. Sánchez J. I. (2016). Efficient breeding by genomic mating. *Front. Genet.* 7:210. 10.3389/fgene.2016.00210 27965707 PMC5126051

[B3] Aliloo H. Pryce J. E. González-Recio O. Cocks B. G. Goddard M. E. Hayes B. J. (2017). Including nonadditive genetic effects in mating programs to maximize dairy farm profitability. *J. Dairy Sci.* 100 1203–1222. 10.3168/jds.2016-11261 27939540

[B4] Allier A. Lehermeier C. Charcosset A. Moreau L. Teyssèdre S. (2019). Improving short and long term genetic gain by accounting for within family variance in optimal cross selection. *Front. Genet.* 10:1006. 10.3389/fgene.2019.01006 31737033 PMC6828944

[B5] Allier A. Teyssèdre S. Lehermeier C. Charcosset A. Moreau L. (2020b). Genomic prediction with a maize collaborative panel: identification of genetic resources to enrich elite breeding programs. *Theor. Appl. Genet.* 133 201–215. 10.1007/s00122-019-03451-9 31595338

[B6] Allier A. Teyssèdre S. Lehermeier C. Moreau L. Charcosset A. (2020a). Optimized breeding strategies to harness Genetic Resources with different performance levels. *BMC Genomics* 21:349. 10.1186/s12864-020-6756-0 32393177 PMC7216646

[B7] Andorf C. Beavis W. D. Hufford M. Smith S. Suza W. P. Wang K. (2019). Technological advances in maize breeding: past, present and future. *Theor. Appl. Genet.* 132 817–849. 10.1007/s00122-019-03306-3 30798332

[B8] Auger D. L. Peters E. M. Birchler J. A. (2005). A genetic test of bioactive gibberellins as regulators of heterosis in maize. *J. Hered.* 96 614–617. 10.1093/jhered/esi102 16135703

[B9] Barrett S. C. H. Charlesworth D. (1991). Effects of a change in the level of inbreeding on the genetic load. *Nature* 352 522–524. 10.1038/352522a0 1865906

[B10] Bar-Zvi D. Lupo O. Levy A. A. Barkai N. (2017). Hybrid vigor: the best of both parents, or a genomic clash? *Curr. Opin. Syst. Biol.* 6 22–27. 10.1016/j.coisb.2017.08.004

[B11] Bernardo R. (1994). Prediction of maize single-cross performance using RFLPs and information from related hybrids. *Crop Sci.* 34 20–25. 10.2135/cropsci1994.0011183x003400010003x

[B12] Bernardo R. (1996). Best linear unbiased prediction of maize single-cross performance given erroneous inbred relationships. *Crop Sci.* 36 862–866. 10.2135/cropsci1996.0011183x003600040007x

[B13] Bernardo R. (2002). *Breeding for Quantitative Traits in Plants*, Vol. 1. Woodbury, MN: Stemma Press, 369.

[B14] Bernardo R. (2009). Genomewide selection for rapid introgression of exotic germplasm in maize. *Crop Sci.* 49 419–425. 10.2135/cropsci2008.08.0452

[B15] Bernardo R. (2020). Reinventing quantitative genetics for plant breeding: something old, something new, something borrowed, something BLUE. *Heredity* 125, 375–385. 10.1038/s41437-020-0312-1 32296132 PMC7784685

[B16] Bernardo R. Yu J. (2007). Prospects for genomewide selection for quantitative traits in maize. *Crop Sci.* 47 1082–1090. 10.2135/cropsci2006.11.0690

[B17] Beukert U. Li Z. Liu G. Zhao Y. Ramachandra N. Mirdita V. (2017). Genome-based identification of heterotic patterns in rice. *Rice* 10 1–10. 28527137 10.1186/s12284-017-0163-4 PMC5438337

[B18] Birchler J. A. Auger D. L. Riddle N. C. (2003). In search of the molecular basis of heterosis. *Plant Cell* 15 2236–2239. 10.1105/tpc.151030 14523245 PMC540269

[B19] Birchler J. A. Yao H. Chudalayandi S. Vaiman D. Veitia R. A. (2010). Heterosis. *Plant Cell* 22 2105–2112. 20622146 10.1105/tpc.110.076133 PMC2929104

[B20] Boeven P. H. Longin C. F. H. Würschum T. (2016). A unified framework for hybrid breeding and the establishment of heterotic groups in wheat. *Theor. Appl. Genet.* 129 1231–1245. 10.1007/s00122-016-2699-x 26956559

[B21] Brauner P. C. Schipprack W. Utz H. F. Bauer E. Mayer M. Schön C. C. (2019). Testcross performance of doubled haploid lines from European flint maize landraces is promising for broadening the genetic base of elite germplasm. *Theor. Appl. Genet.* 132 1897–1908. 10.1007/s00122-019-03325-0 30877313

[B22] Burdo B. Gray J. Goetting−Minesky M. P. Wittler B. Hunt M. Li T. (2014). The Maize TF ome–development of a transcription factor open reading frame collection for functional genomics. *Plant J.* 80 356–366. 10.1111/tpj.12623 25053252 PMC4283594

[B23] Charlesworth D. Charlesworth B. (1987). Inbreeding depression and its evolutionary consequences. *Annu. Rev. Ecol. Syst.* 18 237–268. 10.1146/annurev.es.18.110187.001321

[B24] Charlesworth D. Willis J. H. (2009). The genetics of inbreeding depression. *Nat. Rev. Genet.* 10 783–796. 19834483 10.1038/nrg2664

[B25] Chen Z. J. (2013). Genomic and epigenetic insights into the molecular bases of heterosis. *Nat. Rev. Genet.* 14 471–482. 10.1038/nrg3503 23752794

[B26] Chen Z. J. Birchler J. A. (2013). *Polyploid and Hybrid Genomics.* Hoboken, NJ: John Wiley & Sons.

[B27] Cheverud J. M. Routman E. J. (1995). Epistasis and its contribution to genetic variance components. *Genetics* 139 1455–1461. 10.1093/genetics/139.3.1455 7768453 PMC1206471

[B28] Christensen O. F. Nielsen B. Su G. Xiang T. Madsen P. Ostersen T. (2019). A bivariate genomic model with additive, dominance and inbreeding depression effects for sire line and three-way crossbred pigs. *Genet. Select. Evol.* 51:45. 10.1186/s12711-019-0486-2 PMC6701075 31426753

[B29] Comstock R. E. Robinson H. F. Harvey P. H. (1949). A breeding procedure designed to make maximum use of both general and specific combining ability 1. *Agron. J.* 41 360–367. 10.2134/agronj1949.00021962004100080006x

[B30] Cordell H. J. (2002). Epistasis: what it means, what it doesn’t mean, and statistical methods to detect it in humans. *Hum. Mol. Genet.* 11 2463–2468. 10.1093/hmg/11.20.2463 12351582

[B31] Cowling W. A. Gaynor R. C. Antolín R. Gorjanc G. Edwards S. M. Powell O. (2020). In silico simulation of future hybrid performance to evaluate heterotic pool formation in a self-pollinating crop. *Sci. Rep.* 10 1–8. 10.1002/csc2.20033 32132627 PMC7055256

[B32] Coyne D. P. (1965). Component interaction in relation to heterosis for plant height in *Phaseolus vulgaris* L. Variety crosses 1. *Crop Sci.* 5 17–18. 10.2135/cropsci1965.0011183x000500010007x

[B33] Cress C. E. (1966). A comparison of recurrent selection systems. *Genetics* 54 1371. 10.1093/genetics/54.6.1371 PMC1211302 17248362

[B34] Cros D. Bocs S. Riou V. Ortega-Abboud E. Tisné S. Argout X. (2017). Genomic preselection with genotyping-by-sequencing increases performance of commercial oil palm hybrid crosses. *BMC Genomics* 18:839. 10.1186/s12864-017-4179-3 29096603 PMC5667528

[B35] Cros D. Denis M. Bouvet J. M. Sánchez L. (2015). Long-term genomic selection for heterosis without dominance in multiplicative traits: case study of bunch production in oil palm. *BMC Genomics* 16:651. 10.1186/s12864-015-1866-9 26318484 PMC4552977

[B36] Cros D. Tchounke B. Nkague-Nkamba L. (2018). Training genomic selection models across several breeding cycles increases genetic gain in oil palm in silico study. *Mol. Breed.* 38:89.

[B37] Crow J. F. (1999). “Dominance and overdominance,” in *Genetics and Exploitation of Heterosis in Crops*, eds Coors J. G. Pandey S. (Madison, WI: American Society of Agronomy, Inc), 49–58. 10.2134/1999.geneticsandexploitation.c5

[B38] Crow J. F. (2010). On epistasis: why it is unimportant in polygenic directional selection. *Philos. Trans. R. Soc. B Biol. Sci.* 365 1241–1244. 10.1098/rstb.2009.0275 20308099 PMC2871814

[B39] Daetwyler H. D. Hayden M. J. Spangenberg G. C. Hayes B. J. (2015). Selection on optimal haploid value increases genetic gain and preserves more genetic diversity relative to genomic selection. *Genetics* 200 1341–1348. 10.1534/genetics.115.178038 26092719 PMC4574260

[B40] Dan Z. Hu J. Zhou W. Yao G. Zhu R. Huang W. (2015). Hierarchical additive effects on heterosis in rice (*Oryza sativa* L.). *Front. Plant Sci.* 6:738. 10.3389/fpls.2015.00738 26442051 PMC4566041

[B41] Davenport C. B. (1908). Degeneration, albinism and inbreeding. *Science* 28 454–455. 10.1126/science.28.718.454-b 17771943

[B42] De Beukelaer H. Badke Y. Fack V. De Meyer G. (2017). Moving beyond managing realized genomic relationship in long-term genomic selection. *Genetics* 206 1127–1138. 10.1534/genetics.116.194449 28381589 PMC5499167

[B43] de la Luz Gutiérrez-Nava M. Warren C. A. León P. Walbot V. (1998). Transcriptionally active MuDR, the regulatory element of the mutator transposable element family of Zea mays, is present in some accessions of the Mexican land race *Zapalote chico*. *Genetics* 149 329–346. 9584107 10.1093/genetics/149.1.329 PMC1460140

[B44] de los Campos G. Sorensen D. Gianola D. (2015). Genomic heritability: what is it? *PLoS Genet.* 11:e1005048. 10.1371/journal.pgen.1005048 25942577 PMC4420472

[B45] Dias K. O. G. Piepho H. P. Guimarães L. J. M. Guimarães P. E. O. Parentoni S. N. Pinto M. O. (2019). Novel strategies for genomic prediction of untested single-cross maize hybrids using unbalanced historical data. *Theor. Appl. Genet.* 133 443–455. 10.1007/s00122-019-03475-1 31758202

[B46] Dobzhansky T. (1941). *Genetics and the Origin of Species.* New York, NY: Columbia University Press.

[B47] Dudley J. W. Lambert R. J. (2004). 100 generations of selection for oil and protein in corn. *Plant Breed. Rev.* 24 79–110. 10.1002/9780470650240.ch5

[B48] Duenk P. Calus M. P. Wientjes Y. C. Bijma P. (2017). Benefits of dominance over additive models for the estimation of average effects in the presence of dominance. *G3* 7 3405–3414. 10.1534/g3.117.300113 28842396 PMC5633389

[B49] Duvick D. N. (1999). “Heterosis: feeding people and protecting natural resources,” in *Genetics and Exploitation of Heterosis in Crops*, eds Coors J. G. Pandey S. (Madison, WI: American Society of Agronomy, Inc), 19–29. 10.2134/1999.geneticsandexploitation.c3

[B50] Duvick D. N. (2005). Genetic progress in yield of United States maize (*Zea mays* L.). *Maydica* 50:193.

[B51] Duvick D. N. Smith J. S. C. Cooper M. (2004). Long-term selection in a commercial hybrid maize breeding program. *Plant Breed. Rev.* 24 109–152. 10.1002/9780470650288.ch4

[B52] East E. M. (1908). Inbreeding in corn. *Rep. Conn. Agric. Exp. Stn* 1907 419–428.

[B53] East E. M. (1936). Heterosis. *Genetics* 21:375. 10.1093/genetics/21.4.375 PMC1208682 17246801

[B54] Eynard S. E. Windig J. J. Hulsegge I. Hiemstra S. J. Calus M. P. (2018). The impact of using old germplasm on genetic merit and diversity—A cattle breed case study. *J. Anim. Breed. Genet.* 135 311–322. 10.1111/jbg.12333 29808552

[B55] Falconer D. S. (1985). A note on Fisher’s ‘average effect’and ‘average excess’. *Genet. Res.* 46 337–347. 10.1017/s0016672300022825 4092925

[B56] Falconer D. S. Mackay T. F. C. (1996). *Introduction to Quantitative Genetics.* Essex: Longman Group.

[B57] Fenster C. B. Galloway L. F. Chao L. (1997). Epistasis and its consequences for the evolution of natural populations. *Trends Ecol. Evol.* 12 282–286. 10.1016/s0169-5347(97)81027-0 21238076

[B58] Fernandes S. B. Dias K. O. Ferreira D. F. Brown P. J. (2018). Efficiency of multi-trait, indirect, and trait-assisted genomic selection for improvement of biomass sorghum. *Theor. Appl. Genet.* 131 747–755. 10.1007/s00122-017-3033-y 29218378 PMC5814553

[B59] Fiévet J. B. Nidelet T. Dillmann C. de Vienne D. (2018). Heterosis is a systemic property emerging from non-linear genotype-phenotype relationships: evidence from in vitro genetics and computer simulations. *Front. Genet.* 9:159. 10.3389/fgene.2018.00159 29868111 PMC5968397

[B60] Fischer S. Melchinger A. E. Korzun V. Wilde P. Schmiedchen B. Möhring J. (2010). Molecular marker assisted broadening of the Central European heterotic groups in rye with Eastern European germplasm. *Theor. Appl. Genet.* 120 291–299. 10.1007/s00122-009-1124-0 19669632

[B61] Fisher R. A. (1918). The correlation between relatives on the supposition of mendelian inheritance. *Trans. R. Soc. Edinburgh* 52 399–433. 10.1017/s0080456800012163

[B62] Flint-Garcia S. A. Buckler E. S. Tiffin P. Ersoz E. Springer N. M. (2009). Heterosis is prevalent for multiple traits in diverse maize germplasm. *PLoS One* 4:e7433. 10.1371/journal.pone.0007433 19823591 PMC2757727

[B63] Fort A. Ryder P. McKeown P. C. Wijnen C. Aarts M. G. Sulpice R. (2016). Disaggregating polyploidy, parental genome dosage and hybridity contributions to heterosis in *Arabidopsis thaliana*. *New Phytol.* 209 590–599. 10.1111/nph.13650 26395035

[B64] Fristche-Neto R. Akdemir D. Jannink J. L. (2018). Accuracy of genomic selection to predict maize single-crosses obtained through different mating designs. *Theor. Appl. Genet.* 131 1153–1162. 10.1007/s00122-018-3068-8 29445844

[B65] Fu D. Xiao M. Hayward A. Fu Y. Liu G. Jiang G. (2014). Utilization of crop heterosis: a review. *Euphytica* 197 161–173. 10.1007/s10681-014-1103-7

[B66] Gianinetti A. (2013). A criticism of the value of midparent in polyploidization. *J. Exp. Bot.* 64 4119–4129. 10.1093/jxb/ert263 24006422

[B67] Gianola D. Cecchinato A. Naya H. Schön C. C. (2018). Prediction of complex traits: robust alternatives to best linear unbiased prediction. *Front. Genet.* 9:195. 10.3389/fgene.2018.00195 29951082 PMC6008589

[B68] Gianola D. de los Campos G. Hill W. G. Manfredi E. Fernando R. (2009). Additive genetic variability and the Bayesian alphabet. *Genetics* 183 347–363. 10.1534/genetics.109.103952 19620397 PMC2746159

[B69] Gianola D. Fernando R. L. Stella A. (2006). Genomic-assisted prediction of genetic value with semiparametric procedures. *Genetics* 173 1761–1776. 10.1534/genetics.105.049510 16648593 PMC1526664

[B70] Goddard M. (2009). Genomic selection: prediction of accuracy and maximisation of long term response. *Genetica* 136 245–257. 10.1007/s10709-008-9308-0 18704696

[B71] Goiffon M. Kusmec A. Wang L. Hu G. Schnable P. S. (2017). Improving response in genomic selection with a population-based selection strategy: optimal population value selection. *Genetics* 206 1675–1682. 10.1534/genetics.116.197103 28526698 PMC5500159

[B72] Gorjanc G. Gaynor R. C. Hickey J. M. (2018). Optimal cross selection for long-term genetic gain in two-part programs with rapid recurrent genomic selection. *Theor. Appl. Genet.* 131 1953–1966. 10.1007/s00122-018-3125-3 29876589 PMC6096640

[B73] Govindaraju D. R. (2019). An elucidation of over a century old enigma in genetics—Heterosis. *PLoS Biol.* 17:e3000215. 10.1371/journal.pbio.3000215 31017886 PMC6481769

[B74] Grafius J. E. (1961). The complex trait as a geometric construct. *Heredity* 16 225–228. 10.1038/hdy.1961.24

[B75] Griffing B. (1956a). A generalised treatment of the use of diallel crosses in quantitative inheritance. *Heredity* 10 31–50. 10.1038/hdy.1956.2

[B76] Griffing B. (1956b). Concept of general and specific combining ability in relation to diallel crossing systems. *Aust. J. Biol. Sci.* 9 463–493. 10.1071/bi9560463

[B77] Grindeland J. M. (2008). Inbreeding depression and outbreeding depression in Digitalis purpurea: optimal outcrossing distance in a tetraploid. *J. Evol. Biol.* 21 716–726. 10.1111/j.1420-9101.2008.01519.x 18341541

[B78] Guo T. Yu X. Li X. Zhang H. Zhu C. Flint-Garcia S. (2019). Optimal designs for genomic selection in hybrid crops. *Mol. Plant* 12 390–401. 10.1016/j.molp.2018.12.022 30625380

[B79] Habier D. Fernando R. L. Kizilkaya K. Garrick D. J. (2011). Extension of the Bayesian alphabet for genomic selection. *BMC Bioinform.* 12:186. 10.1186/1471-2105-12-186 21605355 PMC3144464

[B80] Hallauer A. R. Carena M. J. Miranda Filho J. D. (2010). *Quantitative Genetics in Maize Breeding*, Vol. 6. Berlin: Springer Science & Business Media.

[B81] Hayes B. J. Visscher P. M. Goddard M. E. (2009). Increased accuracy of artificial selection by using the realized relationship matrix. *Genet. Res.* 91 47–60. 10.1017/s0016672308009981 19220931

[B82] Heffner E. L. Sorrells M. E. Jannink J. L. (2009). Genomic selection for crop improvement. *Crop Sci.* 49 1–12. 10.1007/978-3-319-63170-7_1

[B83] Henderson C. R. (1975). Best linear unbiased estimation and prediction under a selection model. *Biometrics* 31 423–447. 10.2307/2529430 1174616

[B84] Herbst R. H. Bar-Zvi D. Reikhav S. Soifer I. Breker M. Jona G. (2017). Heterosis as a consequence of regulatory incompatibility. *BMC Biol.* 15:38. 10.1186/s12915-017-0373-7 28494792 PMC5426048

[B85] Heslot N. Jannink J. L. Sorrells M. E. (2015). Perspectives for genomic selection applications and research in plants. *Crop Sci.* 55 1–12. 10.2135/cropsci2014.03.0249

[B86] Hickey J. M. Chiurugwi T. Mackay I. Powell W. Eggen A. Kilian A. (2017). Genomic prediction unifies animal and plant breeding programs to form platforms for biological discovery. *Nat. Genet.* 49:1297. 10.1038/ng.3920 28854179

[B87] Hill W. G. Goddard M. E. Visscher P. M. (2008). Data and theory point to mainly additive genetic variance for complex traits. *PLoS Genet.* 4:e1000008. 10.1371/journal.pgen.1000008 18454194 PMC2265475

[B88] Hochholdinger F. Hoecker N. (2007). Towards the molecular basis of heterosis. *Trends Plant Sci.* 12 427–432. 10.1016/j.tplants.2007.08.005 17720610

[B89] Hölker A. C. Mayer M. Presterl T. Bolduan T. Bauer E. Ordas B. (2019). European maize landraces made accessible for plant breeding and genome-based studies. *Theor. Appl. Genet.* 132 3333–3345. 10.1007/s00122-019-03428-8 31559526 PMC6820615

[B90] Howard R. Gianola D. Montesinos-López O. Juliana P. Singh R. Poland J. (2019). Joint use of genome, pedigree, and their interaction with environment for predicting the performance of wheat lines in new environments. *G3* 9 2925–2934. 10.1534/g3.119.400508 31300481 PMC6723131

[B91] Huang W. Mackay T. F. (2016). The genetic architecture of quantitative traits cannot be inferred from variance component analysis. *PLoS Genet.* 12:e1006421. 10.1371/journal.pgen.1006421 27812106 PMC5094750

[B92] Huang X. Yang S. Gong J. Zhao Q. Feng Q. Zhan Q. (2016). Genomic architecture of heterosis for yield traits in rice. *Nature* 537 629–633. 10.1038/nature19760 27602511

[B93] Huang X. Yang S. Gong J. Zhao Y. Feng Q. Gong H. (2015). Genomic analysis of hybrid rice varieties reveals numerous superior alleles that contribute to heterosis. *Nat. Commun.* 6 1–9. 10.1007/978-3-642-85115-5_1 PMC4327311 25651972

[B94] Ibáñz-Escriche N. Fernando R. L. Toosi A. Dekkers J. C. (2009). Genomic selection of purebreds for crossbred performance. *Genet. Select. Evol.* 41:12. 10.1186/1297-9686-41-12 19284703 PMC2730054

[B95] Jannink J. L. (2010). Dynamics of long-term genomic selection. *Genet. Select. Evol.* 42:35. 10.1186/1297-9686-42-35 PMC2936280 20712894

[B96] Jia Y. Jannink J. L. (2012). Multiple-trait genomic selection methods increase genetic value prediction accuracy. *Genetics* 192 1513–1522. 10.1534/genetics.112.144246 23086217 PMC3512156

[B97] Kacser H. Burns J. A. (1981). The molecular basis of dominance. *Genetics* 97 639–666. 7297851 10.1093/genetics/97.3-4.639 PMC1214416

[B98] Kadam D. C. Lorenz A. J. (2018). “Toward redesigning hybrid maize breeding through genomics-assisted breeding,” in *The Maize Genome*, eds Bennetzen J. Flint-Garcia S. Hirsch C. Tuberosa R. (Cham: Springer), 367–388. 10.1007/978-3-319-97427-9_21

[B99] Kadam D. C. Lorenz A. J. (2019). Evaluation of nonparametric models for genomic prediction of early-stage single crosses in maize. *Crop Sci.* 59 1411–1423. 10.2135/cropsci2017.11.0668

[B100] Kadam D. C. Potts S. M. Bohn M. O. Lipka A. E. Lorenz A. J. (2016). Genomic prediction of single crosses in the early stages of a maize hybrid breeding pipeline. *G3* 6 3443–3453. 10.1534/g3.116.031286 27646704 PMC5100843

[B101] Kaeppler S. (2011). Heterosis: one boat at a time, or a rising tide? *New Phytol.* 189 900–902. 10.1111/j.1469-8137.2010.03630.x 21288243

[B102] Kaeppler S. (2012). Heterosis: many genes, many mechanisms—end the search for an undiscovered unifying theory. *ISRN Bot.* 2012:682824.

[B103] Kemper K. E. Bowman P. J. Pryce J. E. Hayes B. J. Goddard M. E. (2012). Long-term selection strategies for complex traits using high-density genetic markers. *J. Dairy Sci.* 95 4646–4656. 10.3168/jds.2011-5289 22818479

[B104] Kinghorn B. P. Hickey J. M. Van Der Werf J. H. J. (2010). “Reciprocal recurrent genomic selection for total genetic merit in crossbred individuals,” in *Proceedings of the 9th World Congress on Genetics Applied to Livestock Production*, (Leipzig: German Society for Animal Science), 1–6.

[B105] Krieger U. Lippman Z. B. Zamir D. (2010). The flowering gene SINGLE FLOWER TRUSS drives heterosis for yield in tomato. *Nat. Genet.* 42:459. 10.1038/ng.550 20348958

[B106] Lamkey K. R. Edwards J. W. (1999). “Quantitative genetics of heterosis,” in *Genetics and Exploitation of Heterosis in Crops*, eds Coors J. G. Pandey S. (Madison, WI: American Society of Agronomy, Inc), 31–48. 10.2134/1999.geneticsandexploitation.c4

[B107] Lee E. A. Tracy W. F. (2009). “Modern maize breeding,” in *Handbook of Maize*, eds Bennetzen J. L. Hake S. (New York, NY: Springer), 141–160. 10.1007/978-0-387-77863-1_7

[B108] Legarra A. Aguilar I. Misztal I. (2009). A relationship matrix including full pedigree and genomic information. *J. Dairy Sci.* 92 4656–4663. 10.3168/jds.2009-2061 19700729

[B109] Lehner B. (2011). Molecular mechanisms of epistasis within and between genes. *Trends Genet.* 27 323–331. 10.1016/j.tig.2011.05.007 21684621

[B110] Li Z. Zhou P. Della Coletta R. Zhang T. Brohammer A. B. O’Connor C. (2020). Single−parent expression drives dynamic gene expression complementation in maize hybrids. *Plant J.* 10.1111/tpj.15042 [Epub ahead of print]. 33098691

[B111] Liang Z. Gupta S. K. Yeh C. T. Zhang Y. Ngu D. W. Kumar R. (2018). Phenotypic data from inbred parents can improve genomic prediction in pearl millet hybrids. *G3* 8 2513–2522. 10.1534/g3.118.200242 29794163 PMC6027876

[B112] Lippman Z. B. Zamir D. (2007). Heterosis: revisiting the magic. *Trends Genet.* 23 60–66. 10.1016/j.tig.2006.12.006 17188398

[B113] Liu H. Wang Q. Chen M. Ding Y. Yang X. Liu J. (2020). Genome−wide identification and analysis of heterotic loci in three maize hybrids. *Plant Biotechnol. J.* 18 185–194. 10.1111/pbi.13186 31199059 PMC6920156

[B114] Longin C. F. H. Gowda M. Mühleisen J. Ebmeyer E. Kazman E. Schachschneider R. (2013). Hybrid wheat: quantitative genetic parameters and consequences for the design of breeding programs. *Theor. Appl. Genet.* 126 2791–2801. 10.1007/s00122-013-2172-z 23913277

[B115] Longin C. F. H. Mi X. Würschum T. (2015). Genomic selection in wheat: optimum allocation of test resources and comparison of breeding strategies for line and hybrid breeding. *Theor. Appl. Genet.* 128 1297–1306. 10.1007/s00122-015-2505-1 25877519

[B116] Longin C. F. H. Mühleisen J. Maurer H. P. Zhang H. Gowda M. Reif J. C. (2012). Hybrid breeding in autogamous cereals. *Theor. Appl. Genet.* 125 1087–1096. 10.1007/s00122-012-1967-7 22918662

[B117] Longin C. F. H. Reif J. C. Würschum T. (2014). Long-term perspective of hybrid versus line breeding in wheat based on quantitative genetic theory. *Theor. Appl. Genet.* 127 1635–1641. 10.1007/s00122-014-2325-8 24845124

[B118] Lorenz A. J. Chao S. Asoro F. G. Heffner E. L. Hayashi T. Iwata H. (2011). Genomic selection in plant breeding: knowledge and prospects. *Adv. Agron.* 110 77–123.

[B119] Lynch M. (1991). The genetic interpretation of inbreeding depression and outbreeding depression. *Evolution* 45 622–629. 10.2307/2409915 28568822

[B120] Lynch M. Walsh B. (1998). *Genetics and Analysis of Quantitative Traits*, Vol. 1. Sunderland, MA: Sinauer, 535–557.

[B121] Manfredi E. Tusell L. Vitezica Z. G. (2017). Prediction of complex traits: conciliating genetics and statistics. *J. Anim. Breed. Genet.* 134 178–183. 10.1111/jbg.12269 28508479

[B122] Manna F. Martin G. Lenormand T. (2011). Fitness landscapes: an alternative theory for the dominance of mutation. *Genetics* 189 923–937. 10.1534/genetics.111.132944 21890744 PMC3213354

[B123] Marcon C. Schützenmeister A. Schütz W. Madlung J. Piepho H. P. Hochholdinger F. (2010). Nonadditive protein accumulation patterns in maize (*Zea mays* L.) hybrids during embryo development. *J. Proteome Res.* 9 6511–6522. 10.1021/pr100718d 20973536

[B124] Marshall-Colón A. Sengupta N. Rhodes D. Dudareva N. Morgan J. (2010). A kinetic model describes metabolic response to perturbations and distribution of flux control in the benzenoid network of *Petunia hybrida*. *Plant J.* 62 64–76. 10.1111/j.1365-313x.2010.04127.x 20070567

[B125] Marulanda J. J. Mi X. Melchinger A. E. Xu J. L. Würschum T. Longin C. F. H. (2016). Optimum breeding strategies using genomic selection for hybrid breeding in wheat, maize, rye, barley, rice and triticale. *Theor. Appl. Genet.* 129 1901–1913. 10.1007/s00122-016-2748-5 27389871

[B126] Masuka B. Magorokosho C. Olsen M. Atlin G. N. Bänziger M. Pixley K. V. (2017). Gains in maize genetic improvement in Eastern and Southern Africa: II. CIMMYT open-pollinated variety breeding pipeline. *Crop Sci.* 57 180–191. 10.2135/cropsci2016.05.0408

[B127] McKey D. Elias M. Pujol B. Duputié A. (2010). The evolutionary ecology of clonally propagated domesticated plants. *New Phytol.* 186 318–332. 10.1111/j.1469-8137.2010.03210.x 20202131

[B128] Melchinger A. E. (1999). “Genetic diversity and heterosis,” in *Genetics and Exploitation of Heterosis in Crops*, eds Coors J. G. Pandey S. (Madison, WI: American Society of Agronomy, Inc), 99–118. 10.2134/1999.geneticsandexploitation.c10

[B129] Melchinger A. E. Gumber R. K. (1998). “Overview of heterosis and heterotic groups in agronomic crops,” in *Concepts and Breeding of Heterosis in Crop Plants*, Vol. 25 eds Larnkey K. R. Staub J. E. (Madison, WI: Crop Science Society of America, Inc), 29–44. 10.2135/cssaspecpub25.c3

[B130] Melchinger A. E. Singh M. Link W. Utz H. F. Von Kittlitz E. (1994). Heterosis and gene effects of multiplicative characters: theoretical relationships and experimental results from *Vicia faba* L. *Theor. Appl. Genet.* 88 343–348. 10.1007/bf00223643 24186017

[B131] Meuwissen T. H. E. Hayes B. J. Goddard M. E. (2001). Prediction of total genetic value using genome-wide dense marker maps. *Genetics* 157 1819–1829. 11290733 10.1093/genetics/157.4.1819 PMC1461589

[B132] Meuwissen T. H. E. Sonesson A. K. (1998). Maximizing the response of selection with a predefined rate of inbreeding: overlapping generations. *J. Anim. Sci.* 76 2575–2583. 10.2527/1998.76102575x 9814896

[B133] Mikel M. A. Dudley J. W. (2006). Evolution of North American dent corn from public to proprietary germplasm. *Crop Sci.* 46 1193–1205. 10.2135/cropsci2005.10-0371

[B134] Moeinizade S. Hu G. Wang L. Schnable P. S. (2019). Optimizing selection and mating in genomic selection with a look-ahead approach: an operations research framework. *G3* 9 2123–2133. 10.1534/g3.118.200842 31109922 PMC6643893

[B135] Momen M. Mehrgardi A. A. Sheikhi A. Kranis A. Tusell L. Morota G. (2018). Predictive ability of genome-assisted statistical models under various forms of gene action. *Sci. Rep.* 8 1–11. 30120288 10.1038/s41598-018-30089-2 PMC6098164

[B136] Moose S. P. Dudley J. W. Rocheford T. R. (2004). Maize selection passes the century mark: a unique resource for 21st century genomics. *Trends Plant Sci.* 9 358–364. 10.1016/j.tplants.2004.05.005 15231281

[B137] Müller D. Schopp P. Melchinger A. E. (2018). Selection on expected maximum haploid breeding values can increase genetic gain in recurrent genomic selection. *G3* 8 1173–1181. 10.1534/g3.118.200091 29434032 PMC5873908

[B138] Neyhart J. L. Tiede T. Lorenz A. J. Smith K. P. (2017). Evaluating methods of updating training data in long-term genomewide selection. *G3* 7 1499–1510. 10.1534/g3.117.040550 28315831 PMC5427505

[B139] Nyouma A. Bell J. M. Jacob F. Cros D. (2019). From mass selection to genomic selection: one century of breeding for quantitative yield components of oil palm (*Elaeis guineensis* Jacq.). *Tree Genet. Genomes* 15:69.

[B140] Oakley C. G. Ågren J. Schemske D. W. (2015). Heterosis and outbreeding depression in crosses between natural populations of Arabidopsis thaliana. *Heredity* 115 73–82. 10.1038/hdy.2015.18 26059971 PMC4815493

[B141] Okeke U. G. Akdemir D. Rabbi I. Kulakow P. Jannink J. L. (2017). Accuracies of univariate and multivariate genomic prediction models in African cassava. *Genet. Select. Evol.* 49:88. 10.1186/s12711-017-0361-y PMC5715664 29202685

[B142] Paixão T. Barton N. H. (2016). The effect of gene interactions on the long-term response to selection. *Proceedings of the National Academy of Sciences* 113 4422–4427. 10.1073/pnas.1518830113 27044080 PMC4843425

[B143] Phillips P. C. (2008). Epistasis—the essential role of gene interactions in the structure and evolution of genetic systems. *Nat. Rev. Genet.* 9 855–867. 10.1038/nrg2452 18852697 PMC2689140

[B144] Piepho H. P. Möhring J. Melchinger A. E. Büchse A. (2008). BLUP for phenotypic selection in plant breeding and variety testing. *Euphytica* 161 209–228. 10.1007/s10681-007-9449-8

[B145] Pixley K. V. (2006). “Hybrid and open−pollinated varieties in modern agriculture,” in *Plant Breeding: The Arnel R. Hallauer International Symposium*, eds Lamkey K. R. Lee M. (Ames, IA: Blackwell Publishing), 234–250. 10.1002/9780470752708.ch17

[B146] Powers L. (1944). An expansion of Jones’s theory for the explanation of heterosis. *Am. Nat.* 78 275–280. 10.1086/281199

[B147] Ramstein G. P. Jensen S. E. Buckler E. S. (2019). Breaking the curse of dimensionality to identify causal variants in Breeding 4. *Theor. Appl. Genet.* 132 559–567. 10.1007/s00122-018-3267-3 30547185 PMC6439136

[B148] Reif J. C. Gumpert F. M. Fischer S. Melchinger A. E. (2007). Impact of interpopulation divergence on additive and dominance variance in hybrid populations. *Genetics* 176 1931–1934. 10.1534/genetics.107.074146 17507673 PMC1931541

[B149] Reif J. C. Hallauer A. R. Melchinger A. E. (2005). Heterosis and heterotic patterns in maize. *Maydica* 50:215.

[B150] Rembe M. Zhao Y. Jiang Y. Reif J. C. (2019). Reciprocal recurrent genomic selection: an attractive tool to leverage hybrid wheat breeding. *Theor. Appl. Genet.* 132 687–698. 10.1007/s00122-018-3244-x 30488192

[B151] Robertson A. (1960). A theory of limits in artificial selection. *Proc. R. Soc. Lond. B. Biol. Sci.* 153 234–249. 10.1098/rspb.1960.0099

[B152] Rodgers-Melnick E. Bradbury P. J. Elshire R. J. Glaubitz J. C. Acharya C. B. Mitchell S. E. (2015). Recombination in diverse maize is stable, predictable, and associated with genetic load. *Proc. Natl. Acad. Sci. U.S.A.* 112 3823–3828. 10.1073/pnas.1413864112 25775595 PMC4378432

[B153] Rodríguez-Ramilo S. T. García-Cortés L. A. de Cara M. ÁR. (2015). Artificial selection with traditional or genomic relationships: consequences in coancestry and genetic diversity. *Front. Genet.* 6:127. 10.3389/fgene.2015.00127 25904933 PMC4388001

[B154] Rutkoski J. E. Crain J. Poland J. Sorrells M. E. (2017). “Genomic selection for small grain improvement,” in *Genomic Selection for Crop Improvement*, eds Varshney R. K. Roorkiwal M. Sorrells M. E. (Cham: Springer), 99–130. 10.1007/978-3-319-63170-7_5

[B155] Samayoa L. F. Dunne J. C. Andres R. J. Holland J. B. (2018). “Harnessing maize biodiversity,” in *The Maize Genome*, eds Bennetzen J. Flint-Garcia S. Hirsch C. Tuberosa R. (Cham: Springer), 335–366. 10.1007/978-3-319-97427-9_20

[B156] Santantonio N. Jannink J. L. Sorrells M. (2019). Homeologous epistasis in wheat: the search for an immortal hybrid. *Genetics* 211 1105–1122. 10.1534/genetics.118.301851 30679260 PMC6404247

[B157] Santantonio N. Robbins K. R. (2020). A hybrid optimal contribution approach to drive short-term gains while maintaining long-term sustainability in a modern plant breeding program. *bioRxiv [Preprint]* 10.1101/2020.01.08.899039

[B158] Schnable P. S. Springer N. M. (2013). Progress toward understanding heterosis in crop plants. *Annu. Rev. Plant Biol.* 64 71–88. 10.1146/annurev-arplant-042110-103827 23394499

[B159] Schnell F. W. Cockerham C. C. (1992). Multiplicative vs. arbitrary gene action in heterosis. *Genetics* 131 461–469. 10.1093/genetics/131.2.461 1644280 PMC1205018

[B160] Schön C. C. Simianer H. (2015). Resemblance between two relatives–animal and plant breeding. *J. Anim. Breed. Genet.* 132 1–2. 10.1111/jbg.12137 25619306

[B161] Schrag T. A. Schipprack W. Melchinger A. E. (2019). Across-years prediction of hybrid performance in maize using genomics. *Theor. Appl. Genet.* 132 933–946. 10.1007/s00122-018-3249-5 30498894

[B162] Schrag T. A. Westhues M. Schipprack W. Seifert F. Thiemann A. Scholten S. (2018). Beyond genomic prediction: combining different types of omics data can improve prediction of hybrid performance in maize. *Genetics* 208 1373–1385. 10.1534/genetics.117.300374 29363551 PMC5887136

[B163] Schulthess A. W. Zhao Y. Reif J. C. (2017). “Genomic selection in hybrid breeding,” in *Genomic Selection for Crop Improvement*, eds Varshney R. K. Roorkiwal M. Sorrells M. E. (Cham: Springer), 149–183. 10.1007/978-3-319-63170-7_7

[B164] Seifert F. Thiemann A. Schrag T. A. Rybka D. Melchinger A. E. Frisch M. (2018). Small RNA-based prediction of hybrid performance in maize. *BMC Genomics* 19:371. 10.1186/s12864-018-4708-8 29783940 PMC5963143

[B165] Shull G. H. (1908). The composition of a field of maize. *J. Hered.* 4 296–301. 10.1093/jhered/os-4.1.296

[B166] Shull G. H. (1948). What is” heterosis”? *Genetics* 33:439. 10.1093/genetics/33.5.439 PMC1209417 17247290

[B167] Shull G. H. (1952). “Beginnings of a heterosis concept,” in *Heterosis*, ed. Gowen W. (Ames, IA: Iowa State College Press), 14–48.

[B168] Sonesson A. K. Woolliams J. A. Meuwissen T. H. (2012). Genomic selection requires genomic control of inbreeding. *Genet. Select. Evol.* 44:27. 10.1186/1297-9686-44-27 PMC3522025 22898324

[B169] Sprague G. F. Tatum L. A. (1942). General vs. specific combining ability in single crosses of corn 1. *Agron. J.* 34 923–932. 10.2134/agronj1942.00021962003400100008x

[B170] Springer N. M. Stupar R. M. (2007). Allelic variation and heterosis in maize: how do two halves make more than a whole? *Genome Res.* 17 264–275. 10.1101/gr.5347007 17255553

[B171] Stitzer M. C. Ross-Ibarra J. (2018). Maize domestication and gene interaction. *New Phytol.* 220 395–408. 10.1111/nph.15350 30035321

[B172] Stuber C. W. Cockerham C. C. (1966). Gene effects and variances in hybrid populations. *Genetics* 54:1279. 10.1093/genetics/54.6.1279 PMC1211293 17248353

[B173] Sun J. Rutkoski J. E. Poland J. A. Crossa J. Jannink J. L. Sorrells M. E. (2017). Multitrait, random regression, or simple repeatability model in high-throughput phenotyping data improve genomic prediction for wheat grain yield. *Plant Genome* 10 1–12. 10.3835/plantgenome2016.11.0111 28724067

[B174] Swanson-Wagner R. A. Jia Y. DeCook R. Borsuk L. A. Nettleton D. Schnable P. S. (2006). All possible modes of gene action are observed in a global comparison of gene expression in a maize F1 hybrid and its inbred parents. *Proc. Natl. Acad. Sci. U.S.A.* 103 6805–6810. 10.1073/pnas.0510430103 16641103 PMC1447595

[B175] Technow F. (2019). Use of F2 bulks in training sets for genomic prediction of combining ability and hybrid performance. *G3* 9 1557–1569. 10.1534/g3.118.200994 30862623 PMC6505161

[B176] Technow F. Riedelsheimer C. Schrag T. A. Melchinger A. E. (2012). Genomic prediction of hybrid performance in maize with models incorporating dominance and population specific marker effects. *Theor. Appl. Genet.* 125 1181–1194. 10.1007/s00122-012-1905-8 22733443

[B177] Tracy W. F. Chandler M. A. (2006). “The historical and biological basis of the concept of heterotic patterns in corn belt dent maize,” in *Plant Breeding: The Arnel R. Hallauer International Symposium*, eds Lamkey K. R. Lee M. (Ames, IA: Blackwell Publishing), 219–233. 10.1002/9780470752708.ch16

[B178] Trethowan R. M. (2014). “Defining a genetic ideotype for crop improvement,” in *Crop Breeding*, eds Fleury D. Whitford R. (New York, NY: Humana Press), 1–20. 10.1007/978-1-4939-0446-4_1 24816655

[B179] Troyer A. F. (2006). Adaptedness and heterosis in corn and mule hybrids. *Crop Sci.* 46 528–543. 10.2135/cropsci2005.0065

[B180] Troyer A. F. Wellin E. J. (2009). Heterosis decreasing in hybrids: yield test inbreds. *Crop Sci.* 49 1969–1976. 10.2135/cropsci2009.04.0170

[B181] Vacher M. Small I. (2019). Simulation of heterosis in a genome-scale metabolic network provides mechanistic explanations for increased biomass production rates in hybrid plants. *NPJ Syst. Biol. Appl.* 5 1–10. 10.1016/b978-0-12-817953-6.00001-4 31341636 PMC6639380

[B182] Valluru R. Gazave E. E. Fernandes S. B. Ferguson J. N. Lozano R. Hirannaiah P. (2019). Deleterious mutation burden and its association with complex traits in sorghum (*Sorghum bicolor*). *Genetics* 211 1075–1087. 10.1534/genetics.118.301742 30622134 PMC6404259

[B183] van Heerwaarden J. Hufford M. B. Ross-Ibarra J. (2012). Historical genomics of North American maize. *Proc. Natl. Acad. Sci. U.S.A.* 109 12420–12425. 10.1073/pnas.1209275109 22802642 PMC3412004

[B184] VanRaden P. M. (2008). Efficient methods to compute genomic predictions. *J. Dairy Sci.* 91 4414–4423. 10.3168/jds.2007-0980 18946147

[B185] Varona L. Legarra A. Herring W. Vitezica Z. G. (2018a). Genomic selection models for directional dominance: an example for litter size in pigs. *Genet. Select. Evol.* 50:1. 10.1186/s12711-018-0374-1 PMC5787328 29373954

[B186] Varona L. Legarra A. Toro M. A. Vitezica Z. G. (2018b). Non-additive effects in genomic selection. *Front. Genet.* 9:78. 10.3389/fgene.2018.00078 29559995 PMC5845743

[B187] Viana J. M. S. Pereira H. D. Mundim G. B. Piepho H. P. Silva F. F. (2018). Efficiency of genomic prediction of non-assessed single crosses. *Heredity* 120 283–295. 10.1038/s41437-017-0027-0 29180718 PMC5842238

[B188] Vitezica Z. G. Varona L. Elsen J. M. Misztal I. Herring W. Legarra A. (2016). Genomic BLUP including additive and dominant variation in purebreds and F1 crossbreds, with an application in pigs. Genetics Selection Evolution, 48, 1–8. 10.1186/s12711-016-0185-1 26825279 PMC4733284

[B189] Vitezica Z. G. Legarra A. Toro M. A. Varona L. (2017). Orthogonal estimates of variances for additive, dominance, and epistatic effects in populations. *Genetics* 206 1297–1307. 10.1534/genetics.116.199406 28522540 PMC5500131

[B190] Voss-Fels K. P. Cooper M. Hayes B. J. (2019). Accelerating crop genetic gains with genomic selection. *Theor. Appl. Genet.* 132 669–686. 10.1007/s00122-018-3270-8 30569365

[B191] Wallace J. G. Rodgers-Melnick E. Buckler E. S. (2018). On the road to breeding 4.0: unraveling the good, the bad, and the boring of crop quantitative genomics. *Annu. Rev. Genet.* 52 421–444. 10.1146/annurev-genet-120116-024846 30285496

[B192] Wang C. Liu Q. Shen Y. Hua Y. Wang J. Lin J. (2019). Clonal seeds from hybrid rice by simultaneous genome engineering of meiosis and fertilization genes. *Nat. Biotechnol.* 37 283–286. 10.1038/s41587-018-0003-0 30610223

[B193] Wang J. Zhou Z. Zhang Z. Li H. Liu D. Zhang Q. (2018). Expanding the BLUP alphabet for genomic prediction adaptable to the genetic architectures of complex traits. *Heredity* 121 648–662. 10.1038/s41437-018-0075-0 29765161 PMC6221880

[B194] Wang K. Qiu F. Larazo W. dela Paz M. A. Xie F. (2015). Heterotic groups of tropical indica rice germplasm. *Theor. Appl. Genet.* 128 421–430. 10.1007/s00122-014-2441-5 25511903

[B195] Wang X. Li L. Yang Z. Zheng X. Yu S. Xu C. (2017). Predicting rice hybrid performance using univariate and multivariate GBLUP models based on North Carolina mating design II. *Heredity* 118 302–310. 10.1038/hdy.2016.87 27649618 PMC5315526

[B196] Waser N. M. Price M. V. (1994). Crossing−distance effects in *Delphinium nelsonii*: outbreeding and inbreeding depression in progeny fitness. *Evolution* 48 842–852. 10.2307/2410491 28568280

[B197] Washburn J. D. Birchler J. A. (2014). Polyploids as a “model system” for the study of heterosis. *Plant Reprod.* 27 1–5. 10.1007/s00497-013-0237-4 24202960

[B198] Watson A. Hickey L. T. Christopher J. Rutkoski J. Poland J. Hayes B. J. (2019). Multivariate genomic selection and potential of rapid indirect selection with speed breeding in spring wheat. *Crop Sci.* 59 1945–1959. 10.2135/cropsci2018.12.0757

[B199] Wei W. H. Hemani G. Haley C. S. (2014). Detecting epistasis in human complex traits. *Nat. Rev. Genet.* 15 722–733. 10.1038/nrg3747 25200660

[B200] Welch J. J. (2004). Accumulating Dobzhansky−Muller incompatibilities: reconciling theory and data. *Evolution* 58 1145–1156. 10.1554/03-502 15266965

[B201] Werner C. R. Gaynor R. C. Sargent D. J. Lillo A. Gorjanc G. Hickey J. M. (2020). Genomic selection strategies for clonally propagated crops. *bioRxiv [Preprint]* 10.1101/2020.06.15.152017 PMC10036424 36952013

[B202] Werner C. R. Qian L. Voss-Fels K. P. Abbadi A. Leckband G. Frisch M. (2018). Genome-wide regression models considering general and specific combining ability predict hybrid performance in oilseed rape with similar accuracy regardless of trait architecture. *Theor. Appl. Genet.* 131 299–317. 10.1007/s00122-017-3002-5 29080901

[B203] Westhues M. Schrag T. A. Heuer C. Thaller G. Utz H. F. Schipprack W. (2017). Omics-based hybrid prediction in maize. *Theor. Appl. Genet.* 130 1927–1939. 10.1007/s00122-017-2934-0 28647896

[B204] Whittaker J. C. Thompson R. Denham M. C. (2000). Marker-assisted selection using ridge regression. *Genet. Res.* 75 249–252. 10.1017/s0016672399004462 10816982

[B205] Wijnker E. van Dun K. de Snoo C. B. Lelivelt C. L. Keurentjes J. J. Naharudin N. S. (2012). Reverse breeding in *Arabidopsis thaliana* generates homozygous parental lines from a heterozygous plant. *Nat. Genet.* 44:467. 10.1038/ng.2203 22406643

[B206] Williams W. (1959). Heterosis and the genetics of complex characters. *Nature* 184 527–530. 10.1038/184527a0 13844942

[B207] Windhausen V. S. Atlin G. N. Hickey J. M. Crossa J. Jannink J. L. Sorrells M. E. (2012). Effectiveness of genomic prediction of maize hybrid performance in different breeding populations and environments. *G3* 2 1427–1436. 10.1534/g3.112.003699 23173094 PMC3484673

[B208] Woolliams J. A. Berg P. Dagnachew B. S. Meuwissen T. H. E. (2015). Genetic contributions and their optimization. *J. Anim. Breed. Genet.* 132 89–99. 10.1111/jbg.12148 25823835

[B209] Wricke G. Weber E. (eds) (1986). “Hybrid varieties,” in *Quantitative Genetics and Selection in Plant Breeding*, (Berlin: de Gruyter), 257–280.

[B210] Xiang T. Christensen O. F. Vitezica Z. G. Legarra A. (2016). Genomic evaluation by including dominance effects and inbreeding depression for purebred and crossbred performance with an application in pigs. *Genet. Select. Evol.* 48:92. 10.1186/s12711-016-0271-4 PMC5123321 27887565

[B211] Xu Y. Li P. Zou C. Lu Y. Xie C. Zhang X. (2017). Enhancing genetic gain in the era of molecular breeding. *J. Exp. Bot.* 68 2641–2666. 10.1093/jxb/erx135 28830098

[B212] Xue W. Anderson S. N. Wang X. Yang L. Crisp P. A. Li Q. (2019). Hybrid decay: a transgenerational epigenetic decline in vigor and viability triggered in backcross populations of teosinte with maize. *Genetics* 213 143–160. 10.1534/genetics.119.302378 31320409 PMC6727801

[B213] Yang J. Mezmouk S. Baumgarten A. Buckler E. S. Guill K. E. McMullen M. D. (2017). Incomplete dominance of deleterious alleles contributes substantially to trait variation and heterosis in maize. *PLoS Genet.* 13:e1007019. 10.1371/journal.pgen.1007019 28953891 PMC5633198

[B214] Yao H. Gray A. D. Auger D. L. Birchler J. A. (2013). Genomic dosage effects on heterosis in triploid maize. *Proc. Natl. Acad. Sci. U.S.A.* 110 2665–2669. 10.1073/pnas.1221966110 23359717 PMC3574931

[B215] Zhao Y. Li Z. Liu G. Jiang Y. Maurer H. P. Würschum T. (2015a). Genome-based establishment of a high-yielding heterotic pattern for hybrid wheat breeding. *Proc. Natl. Acad. Sci. U.S.A.* 112 15624–15629. 10.1073/pnas.1514547112 26663911 PMC4697414

[B216] Zhao Y. Mette M. F. Reif J. C. (2015b). Genomic selection in hybrid breeding. *Plant Breed.* 134 1–10. 10.1111/pbr.12231

