# Supplementary material for: Heterosis and Hybrid Crop Breeding: A Multidisciplinary Review
Source: Front Genet. 2021 Feb 24;12:643761. doi: 10.3389/fgene.2021.643761 (PMC7943638; doi:10.3389/fgene.2021.643761)
Supplement: Supplementary file 1 [file Table_1.docx]

| **Supplementary Table 1.** Table of equations. |  |  |
| --- | --- | --- |
| **Description of Terms** | **Equation** |  |
| $\sigma_{P}^{2}$ = phenotypic variance $\sigma_{G}^{2}$ = genetic variance $\sigma_{E}^{2}$ = non-genetic variance $\sigma_{GE}^{2}$ = interaction variance | $\sigma_{P}^{2} = \sigma_{G}^{2}+ \sigma_{E}^{2}+ \sigma_{GE}^{2}$ | (Eq. 1) |
| $\sigma_{G}^{2}$ = genetic variance $\sigma_{A}^{2}$ = additive variance $\sigma_{D}^{2}$ = dominance variance $\sigma_{I}^{2}$ = epistatic variance | $\sigma_{G}^{2} = \sigma_{A}^{2}+ \sigma_{D}^{2}+ \sigma_{I}^{2}$ | (Eq. 2) |
| $M$ = population mean $a$ = genetic value of genotype *jj* $p$ = frequency of allele *h* $q$ = frequency of allele *j* $d$ = genetic value of genotype *hj* | $M=a\left( p-q \right)+2dpq$ | (Eq. 3)* |
| $\alpha_{h}$ = average effect of allele *h* $q$ = frequency of allele *j* $a$ = genetic value of genotype *jj* $d$ = genetic value of genotype *hj* $p$ = frequency of allele *h* | $\alpha_{h}=q\left[ a+d\left( q-p \right) \right]$ | (Eq. 4)* |
| $\alpha_{j}$ = average effect of allele *j* $p$ = frequency of allele *h* $a$ = genetic value of genotype *jj* $d$ = genetic value of genotype *hj* $q$ = frequency of allele *j* | $\alpha_{j}= -p\left[ a+d\left( q-p \right) \right]$ | (Eq. 5)* |
| **Supplementary Table 1 (cont.)** |  |  |
| **Description of Terms** | **Equation** |  |
| $G_{yz}$ = genetic value of genotype *h_y_j­_z_* $\mu_{G}$ = population mean genetic value $\alpha_{h}$ = average effect of allele *h* $N_{h}$ = number of copies of allele *h* in genotype *h_y_j_z_* $\alpha_{j}$ = average effect of allele *j* $N_{j}$ = number of copies of allele *j* in genotype *h_y_j_z_* $\delta_{hj}$ = dominance deviation of genotype *h_y_j_z_* | $G_{yz}= \mu_{G}+ \alpha_{h}N_{h}+ \alpha_{j}N_{j}+ \delta_{hj}$ | (Eq. 6) |
| $G_{ijkl}$ = genetic value of individual with alleles *i* and *j* at one locus and alleles *k* and *l* at another locus $\mu_{G}$ = population mean genetic value $\alpha_{i}$ = average effect of allele *i* $\alpha_{j}$ = average effect of allele *j* $\alpha_{k}$ = average effect of allele *k* $\alpha_{l}$ = average effect of allele *l*$\delta_{ij}$ = dominance deviation of genotype *ij* $\delta_{kl}$ = dominance deviation of genotype *kl* $(\alpha\alpha)_{ik}$ = epistatic additive x additive effect of allele *i* and allele *k* $(\alpha\alpha)_{il}$ = epistatic additive x additive effect of allele *i* and allele *l* $(\alpha\alpha)_{jk}$ = epistatic additive x additive effect of allele *j* and allele *k* $(\alpha\alpha)_{jl}$ = epistatic additive x additive effect of allele *j* and allele *l* $(\alpha\delta)_{ikl}$ = epistatic additive x dominance effect of allele *i* with genotype *kl* $(\alpha\delta)_{jkl}$ = epistatic additive x dominance effect of allele *j* with genotype *kl* $(\alpha\delta)_{ijk}$ = epistatic additive x dominance effect of allele *k* with genotype *ij* $(\alpha\delta)_{ijl}$ = epistatic additive x dominance effect of allele *l* with genotype *ij* $(\delta\delta)_{ijkl}$ = dominance x dominance effect of genotype *ij* with genotype *kl* … = terms implied by extension to additional loci | $G_{ijkl}= \mu_{G}+\left[ \alpha_{i}+\alpha_{j}+\alpha_{k}+\alpha_{l} \right]+\left[ \delta_{ij}+ \delta_{kl} \right]+[(\alpha\alpha)_{ik}+(\alpha\alpha)_{il}+(\alpha\alpha)_{jk}+\left( \alpha\alpha)_{jl} \right]+[(\alpha\delta)_{ikl}+(\alpha\delta)_{jkl}+(\alpha\delta)_{ijk}+\left( \alpha\delta)_{ijl} \right]+(\delta\delta)_{ijkl}+\ldots$ | (Eq. 7) |
| **Supplementary Table 1 (cont.)** |  |  |
| **Description of Terms** | **Equation** |  |
| $MPH$ = mid-parent heterosis  $\mu_{F1}$ = genetic value of F_1_ progeny $\mu_{P1}$ = genetic value of Parent 1  $\mu_{P2}$ = genetic value of Parent 2 | $MPH= \mu_{F1}- \frac{\mu_{P1}+ \mu_{P2}}{2}$ | (Eq. 8) |
| $\bar{F_{1}}$ = population mean of the F_1_ generation  $p$ = frequency of allele *h* in subpopulation A  $q$ = frequency of allele *j* in subpopulation A  $y$ = difference in allele frequency between subpopulations A and B  $a$ = genetic value of genotype *jj*  $d$ = genetic value of genotype *hj* | $\bar{F_{1}}=\left( p-q-y \right)a+\left[ 2pq+y\left( p-q \right) \right]d$ | (Eq. 9)** |
| $PMPV$ = panmictic-midparent value  $p$ = frequency of allele *h* in subpopulation A  $q$ = frequency of allele *j* in subpopulation A  $y$ = difference in allele frequency between subpopulation A and B  $a$ = genetic value of genotype *jj*  $d$ = genetic value of genotype *hj* | $PMPV= \left( \text{p}-\text{q}-y \right)a+\left[ 2\text{pq}+y\left( \text{p}-\text{q} \right)-y^{2} \right]d$ | (Eq. 10)** |
| $PMPH$ = panmictic midparent heterosis  $y$ = difference in allele frequency between subpopulations A and B  $d$ = genetic value of genotype *hj* | $PMPH= y^{2}d$ | (Eq. 11)** |
| **Supplementary Table 1 (cont.)** |  |  |
| **Description of Terms** | **Equation** |  |
| $F_{2}H$ = F_2_ heterosis  $y$ = difference in allele frequency between subpopulations A and B  $d$ = genetic value of genotype *hj* | $F_{2}H= {\frac{1}{2}y}^{2}d$ | (Eq. 13)** |
| $BH$ = baseline heterosis  $\bar{p}$ = the average allele frequency of allele *h* in the cross of two subpopulations  $\bar{q}$ = the average allele frequency of allele *j* in the cross of two subpopulations  $d$ = genetic value of genotype *hj*  $y$ = difference in allele frequency between parents | $BH=2\bar{p}\bar{q}d- \frac{1}{2}yd^{2}$ | (Eq. 14)** |
| $IMPH$ = inbred-midparent heterosis  $\bar{p}$ = the average allele frequency of allele *h* in the cross of two subpopulations  $\bar{q}$ = the average allele frequency of allele *j* in the cross of two subpopulations  $d$ = genetic value of genotype *hj*  $y$ = difference in allele frequency between parents | $IMPH=2\bar{p}\bar{q}d+ \frac{1}{2}yd^{2}$ | (Eq. 15)** |
| $IMPV$ = the inbred-midparent value  $p$ = frequency of allele *h* in subpopulation A  $q$ = frequency of allele *j* in subpopulation A  $y$ = difference in allele frequency between subpopulations A and B  $a$ = genetic value of genotype *jj* | $IMPV=\left( \text{p}-\text{q}-y \right)a$ | (Eq. 16)** |
| **Supplementary Table 1 (cont.)** |  |  |
| **Description of Terms** | **Equation** |  |
| $\bar{P}$ = mid-parent phenotypic value $b_{0}$ = intercept; grand mean of offspring phenotypic value $b_{1}$ = slope; estimates narrow-sense heritability *O* = offspring phenotypic value $\varepsilon$ = residual error | $\bar{P}=b_{0}+b_{1}O+ \varepsilon$ | (Eq. 17) |
| $Y$ = vector of phenotypic values $\mathbf{X}$ = incidence matrix of fixed effects $\beta$ = vector of fixed effects $\mathbf{Z}$ = incidence matrix of random effects $u$ = vector of random effects (e.g. breeding values) $\varepsilon$ = vector of residual errors | $Y=\mathbf{X}\beta+\mathbf{Z}u+\varepsilon$ | (Eq. 18) |
| $Y$ = vector of phenotypic values $\mathbf{W}$ = incidence matrix $\mathbf{G}$ = additive marker genotype matrix $u$ = vector of marker effects $\varepsilon$ = residual error | $Y=\mathbf{WG}u\boldsymbol{+}\varepsilon$ | (Eq. 19) |
| $v_{ij}$ = phenotypic value for hybrid of parents *i* and *j* $g_{i}$ = general combining ability of parent *i* $g_{j}$ = general combining ability of parent *j* $s_{ij}$ = specific combining ability of parent combination *ij* | $v_{ij}= g_{i}+ g_{j}+ s_{ij}$ | (Eq. 20) |

*Assumes a random-mating population in Hardy-Weinburg equilibrium
** From Lamkey & Edwards, 1999, but formulated in the notation of Falconer & Mackay, 1996, by R. Chris Gaynor.
